# T and B Lymphocyte Transcriptional States Differentiate between Sensitized and Unsensitized Individuals in Alpha-Gal Syndrome

**DOI:** 10.3390/ijms22063185

**Published:** 2021-03-20

**Authors:** Onyinye I. Iweala, Shailesh K. Choudhary, Claire T. Addison, Scott P. Commins

**Affiliations:** 1 Department of Pediatrics, University of North Carolina Food Allergy Initiative, Division of Allergy, Immunology and Rheumatology, University of North Carolina at Chapel Hill, Chapel Hill, NC 27599, USA; shailesh_choudhary@med.unc.edu (S.K.C.); clairea@live.unc.edu (C.T.A.); scommins@email.unc.edu (S.P.C.); 2 Thurston Arthritis Research Center, Division of Rheumatology, Allergy, and Immunology, Department of Medicine, University of North Carolina at Chapel Hill, Chapel Hill, NC 27599, USA

**Keywords:** alpha-gal syndrome, red meat allergy, alpha-gal, tick, IgE

## Abstract

The mechanisms of pathogenesis driving alpha-gal syndrome (AGS) are not fully understood. Differences in immune gene expression between AGS individuals and non-allergic controls may illuminate molecular pathways and targets critical for AGS development. We performed immune expression profiling with RNA from the peripheral blood mononuclear cells (PBMCs) of seven controls, 15 AGS participants, and two participants sensitized but not allergic to alpha-gal using the NanoString nCounter PanCancer immune profiling panel, which includes 770 genes from 14 different cell types. The top differentially expressed genes (DEG) between AGS subjects and controls included transcription factors regulating immune gene expression, such as the NFκB pathway (*NFKBIA, NFKB2, REL*), antigen presentation molecules, type 2/allergic immune responses, itch, and allergic dermatitis. The differential expression of genes linked to T and B cell function was also identified, including transcription factor *BCL-6*, markers of antigen experience (*CD44*) and memory (*CD27*), chemokine receptors (*CXCR3, CXCR6*), and regulators of B-cell proliferation, cell cycle entry and immunoglobulin production (*CD70*). The PBMCs from AGS subjects also had increased TNF and IFN-gamma mRNA expression compared to controls. AGS is associated with a distinct gene expression profile in circulating PBMCs. DEGs related to antigen presentation, antigen-experienced T-cells, and type 2 immune responses may promote the development of alpha-gal specific IgE and the maintenance of AGS.

## 1. Introduction

Alpha-gal syndrome (AGS) refers to the allergic symptoms generated by immune-mediated hypersensitivity responses to the glycan galactose-alpha-1,3-galactose (alpha-gal). It encompasses two classic phenotypes, namely, immediate hypersensitivity responses to injected pharmaceutical products that contain alpha-gal, and delayed allergic reactions following oral ingestion of red meat (i.e., mammalian food products) [1,2]. Alpha-gal mammalian meat allergy challenges the current paradigm for food allergy because, in contrast to conventional food allergies, symptom onset after alpha-gal consumption is delayed, occurring 2 h or longer after ingestion. In addition, in the US, sensitization to alpha-gal and the appearance of circulating alpha-gal IgE antibodies is associated with bites from the lone star tick (*Amblyomma americanum*), while bites from other tick species have been linked to the development of AGS in other countries around the globe [3,4].

Both alpha-gal-specific (sIgE) and total IgE have been shown to increase in human subjects after bites from adult, larval, or nymphal-stage ticks [5]. Moreover, alpha-gal sugar is detectable in ticks associated with alpha-gal syndrome [3,6,7]. For example, alpha-gal was detected in the saliva and salivary glands of the lone star tick [8]. Yet, the mechanisms of pathogenesis behind AGS, including how tick bites from select species lead to a rise in alpha-gal sIgE and which host characteristics favor the development of the clinical symptoms associated with AGS, have not been fully elucidated.

To begin to address this gap, we compared the expression of genes associated with immune function among alpha-gal-sensitized and allergic individuals and non-allergic controls. To that end, we harvested peripheral blood mononuclear cells (PBMCs) from alpha-gal-sensitized subjects, with and without clinical AGS, and from control subjects who were neither sensitized nor allergic to alpha-gal, and performed immune gene expression profiling using the NanoString PanCancer immune profiling panel.

In murine models of tick infestation, it has been shown that tick infestation or the injection of tick saliva can promote the development of T-helper 2 (Th2) immune responses [9,10,11]. Thus, we hypothesized that lone star tick bites drove sensitization to alpha-gal by injecting alpha-gal into the host during the biting process, while concurrently pushing a Th2 immune response typically associated with clearing parasite infection or infestation. Thus, we expected that individuals in our cohort sensitized to alpha-gal after a lone star tick bite would exhibit an immune gene expression profile that was skewed toward type 2 immunity.

## 2. Results

### 2.1. Subject Characteristics

**Figure 1:**
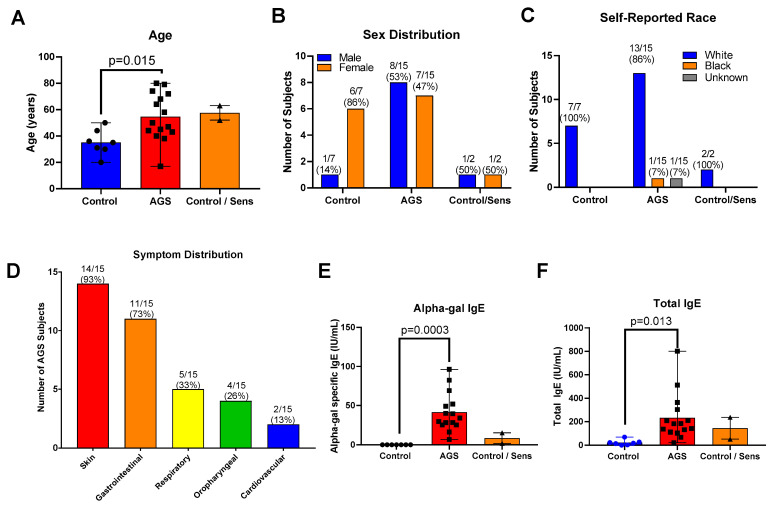
Study participant characteristics for multiplex gene expression analysis cohort. (**A**) Age. (**B**) Sex. (**C**) Self-reported race. (**D**) Symptom distribution. (**E**) Serum alpha-gal-specific IgE. (F) Serum total IgE. Control (*n* = 7); sensitized but asymptomatic (“Control/Sens,” *n* = 2); Alpha-gal syndrome (“AGS”; *n* = 15). *p* values were generated using an unpaired *t* test comparing AGS to control, with *p* < 0.05 considered significant. Due to the small sample size, the sensitized but asymptomatic group was excluded from the statistical analysis.

We recruited 40 total participants for this study (Tables S1 and S2). Of these, 21 provided peripheral blood mononuclear cells (PBMCs) for the multiplex gene expression analysis only; 16 provided PBMCs for flow cytometry only, and 3 participants provided PBMCs for both analyses. Of the 24 total participants who provided PBMCs for the transcriptional analysis portion of this study, 7 were controls who were neither sensitized nor allergic to alpha-gal. Of these, 2 were sensitized to alpha-gal, with alpha-gal IgE level ≥0.10 kU/L but no history of allergic reaction following mammalian meat ingestion (referred to as “sensitized” or “C/S”), and 15 participants had alpha-gal syndrome (sensitized with alpha-gal IgE level ≥ 0.1 kU/L and a history of allergic reaction after ingesting alpha-gal, referred to as “AGS”). In the gene expression analysis cohort, those sensitized to alpha-gal were older than the control participants (the mean age of sensitized subjects was 57.5 years (range 52–63); the mean age of AGS participants was 54.6 years (range 17–80 years); the mean age of control participants was 35.1 years (range 20–50 years); Table S2 and Figure 1A). There was a higher proportion of men in the AGS group (53%) than among the controls (14%, Table S2 and Figure 1B). The participants were predominantly white (Table S2, Figure 1C). The majority of participants with AGS reported cutaneous (93% or 14/15 participants) and gastrointestinal symptoms (73% or 11/15 participants) (Table S1 and Figure 1D), as has been described previously in other cohorts [12,13]. One-third or less reported respiratory, oropharyngeal, or cardiovascular symptoms following the ingestion of alpha-gal. As expected, sensitized and AGS participants had higher levels of alpha-gal-specific IgE than controls, in the range of 6.75 to 96.4 IU/mL (Table S2 and Figure 1E). AGS participants also had higher levels of total IgE compared to controls (Figure 1F).

### 2.2. Differential Gene Expression in Peripheral Blood Mononuclear Cells from Controls and Alpha-Gal-Sensitized Participants

**Figure 2:**
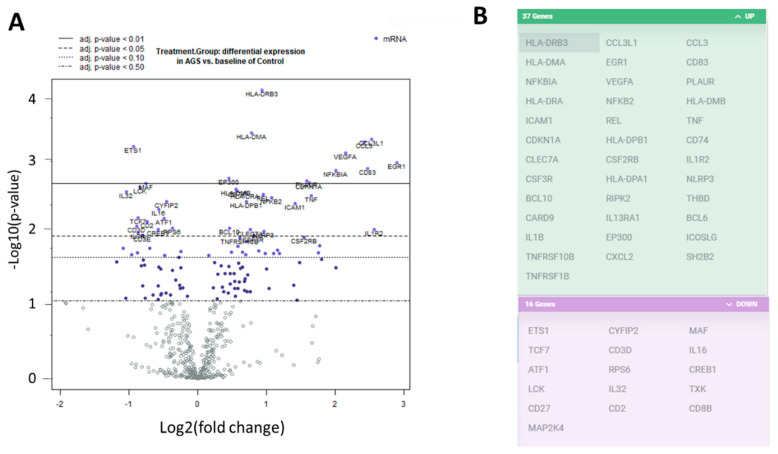
Differentially expressed genes in peripheral blood mononuclear cells (PBMCs) from alpha-gal allergic participants compared to controls. (**A**) Volcano plots show −log10(*p*-value) and Log 2 fold change in gene expression for AGS participants compared to control. Highly statistically significant genes are at the top of the plot above the horizontal lines, which represent the *p*-value thresholds adjusted using the Benjamini–Hochberg method of estimating false discovery rates (FDR). Highly differentially expressed genes fall to the left and right. There are 81 genes wherein the adjusted *p*-value for differential gene expression is *p* < 0.05, and 53 genes wherein the adjusted *p*-value is *p* < 0.02. The 40 most statistically significant genes are labeled in the plot. (**B**) The graphic lists the 53 genes with adjusted *p*-value < 0.02. The 37 gene transcripts that are highlighted in green were upregulated, and the 16 gene transcripts highlighted in purple were downregulated, in AGS compared to control.

We investigated the transcriptional profiles of the PBMCs from the alpha-gal-sensitized individuals and the unsensitized, non-allergic control individuals. We extracted RNA from PBMCs and used the digital multiplexed NanoString^®^ nCounter analysis system to perform gene expression profiling. We analyzed our RNA samples using the nCounter PanCancer immune profile panel, which incorporated 770 human genes—40 reference (housekeeping genes) and 730 immune genes. Volcano plots showing the −log10 (*p*-value) vs. Log 2 fold change in gene expression for the AGS participants compared to the control are shown in Figure 2A. There were 81 genes wherein the absolute fold change between AGS and controls was greater than or equal to 1.3, with an adjusted *p*-value < 0.05, determined using the Benjamini–Hochberg method of estimating false discovery rates (FDR). A further 53 genes had an absolute fold change of greater than or equal to 1.3, with an adjusted *p*-value < 0.02. Gene expression for 37 of those 53 genes was upregulated in AGS compared to controls, and downregulated for 16 of 53 (Figure 2B and Table S3). In total, 12 DEGs, including *ILR2*, were associated with the innate immune response, and 23 DEGs were associated with T or B cell function in either nCounter PanCancer immune profiling annotation or the UniProt gene ontology biological process annotation (Table S3 and www.uniprot.org, accessed 7 March 2021). When compared to control subjects, AGS participants showed an increased expression of genes associated with the NFkappaB pathway, and the transcriptional regulation of immune genes, antigen processing and presentation, type 2/Th2 immune responses, itch, and allergic dermatitis (Table S3).

**Figure 3:**
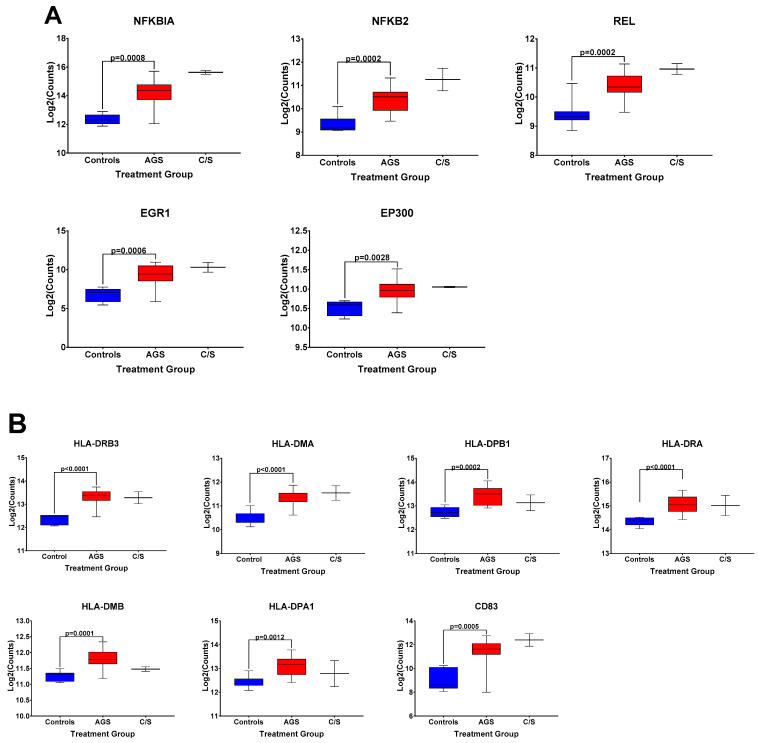

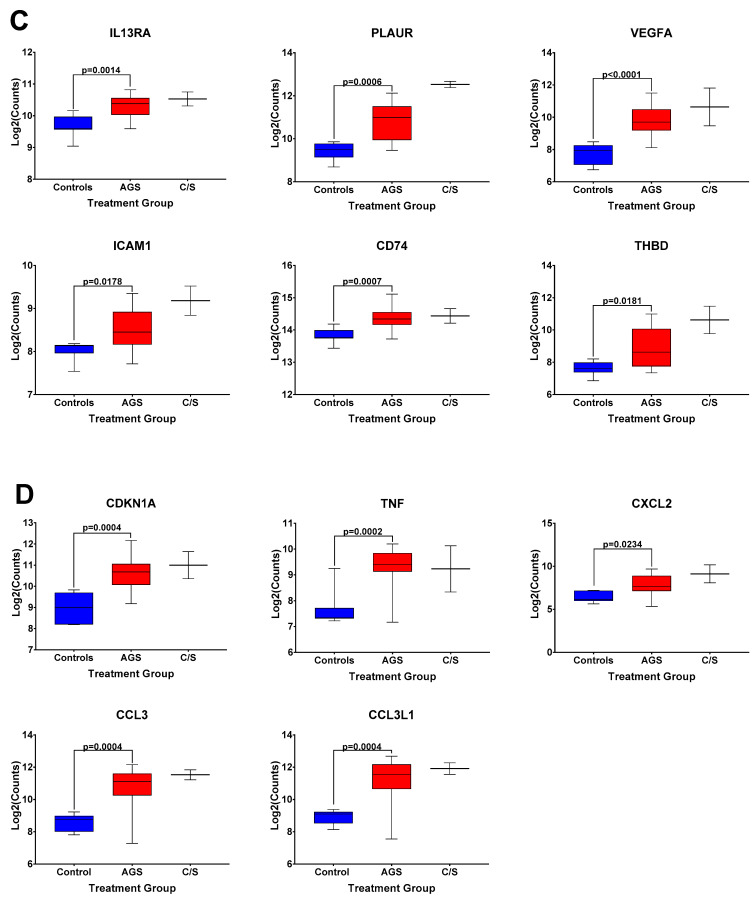
Normalized expression levels of selected immune response-related genes upregulated in PBMCs from AGS subjects compared to controls. (**A**) Genes involved in transcriptional regulation and/or the NFkappaB pathway. (**B**) Genes associated with antigen presentation and MHC II surface expression. (**C**) Genes associated with type 2/Th2 allergic immune responses. (**D**) Genes associated with itch and allergic dermatitis. *p* values were generated using an unpaired *t* test comparing AGS to controls, with *p* < 0.05 considered significant. Due to the small sample size, the sensitized but asymptomatic (“C/S”) group was excluded from statistical analysis. AGS = alpha-gal syndrome.

The box and whisker plots of normalized gene expression counts for NFKB family transcription factors (Figure 3A), MHC II molecules and cell surface molecules instrumental for antigen presentation (Figure 3B), cytokines, growth factors, and adhesion molecules associated with type 2 immune responses (Figure 3C), and genes associated with itch and atopic dermatitis (Figure 3D), showed that AGS participants exhibited an increased transcription of genes in these functional categories when compared to controls. Due to the small sample size (*n* = 2) and significant difficulty recruiting more of these subjects in the setting of the COVID-19 pandemic, the alpha-gal -sensitized but asymptomatic group (C/S) was excluded from statistical analysis. However, the patterns of differential gene expression (Figure S1) and the normalized expression levels of genes within those immune response categories in PBMCs from these participants followed the same trends as in the AGS group (Figure 3). With the caveat that the sample size for the C/S group was small, expression levels were upregulated for 21 identical genes and downregulated for 7 in AGS and C/S PBMCs when either group was compared to controls (Figure S2). When gene expression was compared between AGS and the sensitized but asymptomatic groups, there were no DEGs for which the adjusted *p*-value was less than 0.05 (Table S4, adjusted *p*-values are in bold).

### 2.3. Transcriptional Immune Profiles for T and B Cell Function in PBMCs from Control and Alpha-Gal-Sensitized Subjects Are Largely Distinct

**Figure 4:**
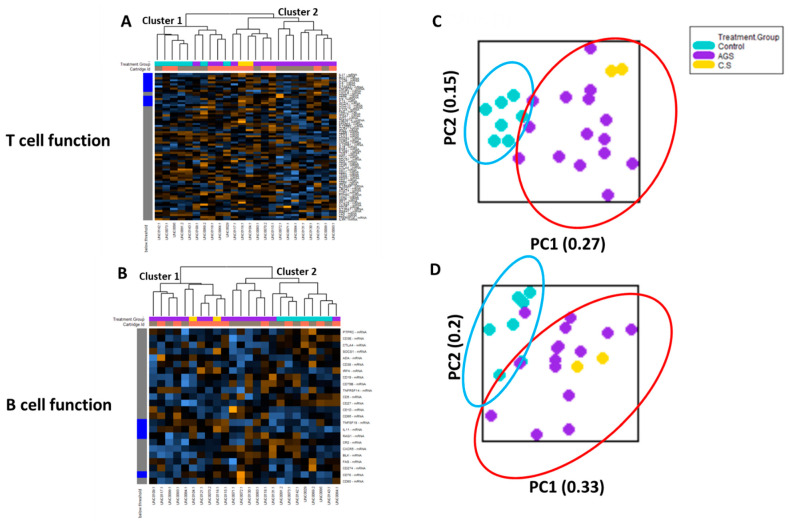
Transcriptional immune profiles for B and T cell function from control and alpha-gal-sensitized subjects are largely distinct. Heatmap of the normalized gene expression data and unsupervised hierarchical clustering of genes associated with (**A**) T cell function and (**B**) B cell function. (**C**) Principal component analysis of gene expression data associated with T cell function and (**D**) B cell function. Control (*n* = 7); sensitized (“CS” *n* = 2); alpha-gal syndrome (“AGS” *n* = 15).

Cell type profiling across all 24 samples using the nCounter Advanced Analysis software algorithm (which uses genes on the panel that are characteristic of various cell populations to measure relative cell population abundance) demonstrated that in the PBMC fractions from study participants, lymphocytes predominated, including adaptive (T and B) lymphocytes (Figure S3). Since T and B lymphocytes are critical for the sensitization phase in conventional food allergies [14], we performed an unsupervised hierarchical clustering of the genes associated with T cell function and B cell function, respectively (Figure 4). The heat map generated showed that the participants were separated into two major clusters based on T cell-associated immune gene expression. One cluster (“Cluster 2”) included only alpha-gal-sensitized participants (12 AGS and 2 sensitized but asymptomatic participants). The other cluster (“Cluster 1”) included all seven controls and three AGS participants (Figure 4A). The unsupervised hierarchical clustering of genes associated with B cell function showed that participants were also separated into two major clusters based on B cell-associated immune gene expression. One cluster (“Cluster 1”) included only alpha-gal-sensitized subjects, including 8 of the 15 AGS participants and the 2 sensitized but asymptomatic participants. The second cluster (“Cluster 2”) included all 7 controls, and 7 of 15 AGS participants. Within “Cluster 2,” one subcluster contained only AGS participants (5 of 15), while the second included 2 out of the 15 AGS and all 7 control participants (Figure 4B).

The principal component (PC) analysis of data on the T cell function gene sets showed that participants sensitized to alpha-gal (i.e., “C/S” and “AGS”) clustered together. The control subjects also clustered together. The PC plots for the control (teal oval) and alpha-gal-sensitized (red oval) clusters did overlap, with 3 out of 17 total alpha-gal-sensitized participants clustering close to the controls (Figure 4C). PC analysis of the data from the B cell function gene sets showed that with the exception of two AGS participants, the participants sensitized to alpha-gal (i.e., “C/S” and “AGS”) clustered together. The 7 controls also clustered together and overlapped with 2 out of the 17 total alpha-gal-sensitized participants (Figure 4D).

### 2.4. Differential Expression of Genes in PBMCs Linked to Invariant Natural Killer T (iNKT) Cell Development and Function

The IgG and IgM antibodies against alpha-gal generated by human B cells depend in part on CD1d-mediated interactions between B cells and invariant (i) natural killer T (NKT) cells [15]. In addition, iNKT cells are capable of producing type 2 cytokines, such as IL-4, that push B cells to class switch into IgE [16]. Thus, we examined circulating iNKT cells from a second cohort of AGS (*n* = 11) and control subjects (*n* = 8, see participant characteristics in Tables S1 and S2). The circulating iNKT cells were identified by flow cytometry as CD3^+^hCD1d-PBS57^+^, i.e., CD3-expressing T cells that also bound to human CD1d tetramer complexed with PBS57, a glycosphingolipid analog of the canonical iNKT cell ligand alpha-galactosylceramide [17]. We found that CD1d-restricted iNKT cells are a rare cell population detectable in the peripheral blood of both control and alpha-gal allergic subjects (Figure S4). The median frequency of circulating iNKT cells in controls was 0.017%, while the median frequency in AGS subjects was 0.013% (Table S2). The median frequency of circulating activated CD69^+^ iNKT cells in AGS subjects at baseline (e.g., no reported recent tick bite; no recent mammalian meat ingestion) was 17.2%, which is 2.5 times greater than the median frequency of activated CD69^+^ iNKT cells in control PBMCs (6.38%), but it did not reach statistical significance (Table S2 and Figure S4D). There was a weak positive linear correlation between the proportion of CD3^+^hCD1d-PBS57^+^ iNKT cells among PBMCs and alpha-gal-sIgE levels (R^2^ = 0.34, *p* = 0.01). There was also a weak positive linear correlation between the proportion of CD69^+^ iNKT cells among PBMCs and the alpha-gal-sIgE levels that did not reach statistical significance (R^2^ = 0.21, *p* = 0.053, using Pearson correlation coefficient calculation with a two-tailed *p*-value and a 95% confidence interval). The median frequency of CD3^+^ non-iNKT cells was comparable across controls and AGS subjects (50.25% vs. 48.20%), as was the median frequency of CD69^+^ among the CD3^+^non-iNKT cells (0.92% vs. 1.05%, controls vs. AGS, Table S2 and Figure S4C,E).

**Figure 5:**
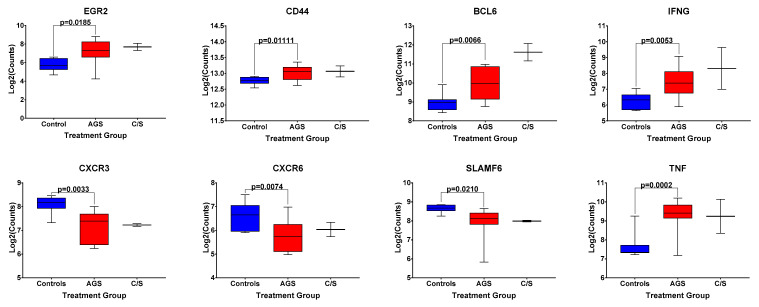
Normalized expression levels of select genes associated with iNKT cell development and effector function in bulk PBMCs from control, alpha-gal-sensitized but asymptomatic, and AGS subjects. *p* values were generated using an unpaired *t* test comparing AGS to controls, with *p* < 0.05 considered statistically significant. Due to the small sample size, the sensitized but asymptomatic group was excluded from statistical analysis.

Because of this trend toward an increased frequency of circulating, activated CD1d-restricted iNKT cells in AGS subjects, compared to the controls in this second cohort, we returned to data from our original cohort of alpha-gal-sensitized and unsensitized control subjects, and examined the differential expression of genes associated with iNKT cell development and effector function [18] included within the NanoString Human PanCancer panel (Figure 5). Of note, gene expression analysis was performed on bulk PBMCs, and all the genes presented in Figure 5 are also associated with the function of non-iNKT T and B cells, cell types that are more abundant than iNKT cells in human peripheral blood (Figures S3 and S4). A comparison among AGS subjects and unsensitized controls in our original cohort showed the statistically significantly upregulated expression of *BCL-6*, a transcription factor that regulates the conventional T and iNKT follicular helper cell subsets [19] and is also expressed in germinal center B cells [20]. The gene expression of *CD44*, a marker of antigen experience, and the cytokines *IFNG* and *TNF*, both associated with iNKT cell function [16], in addition to conventional T cell function, were also significantly upregulated (Figure 5 and Table S3). Transcripts of the chemokine receptors *CXCR3* and *CXCR6* and the transcription factor *SLAMF6* were significantly downregulated in AGS compared to controls. Expression of *EGR2*, which, like *SLAMF6*, is a transcription factor whose expression has been associated with iNKT cell development [19], was upregulated in AGS (Figure 5). Although the alpha-gal-sensitized but asymptomatic group was excluded from the statistical analysis because of the small sample size, the expression patterns for *EGR2*,* CD44*,* BCL6*,* IFNG*,* CXCR3*,* CXCR6*,* SLAMF6*, and *TNF* resembled those of the AGS group (Figure 5).

### 2.5. Differential Expression of Genes Linked to B Cell Function in AGS Participants

**Figure 6:**
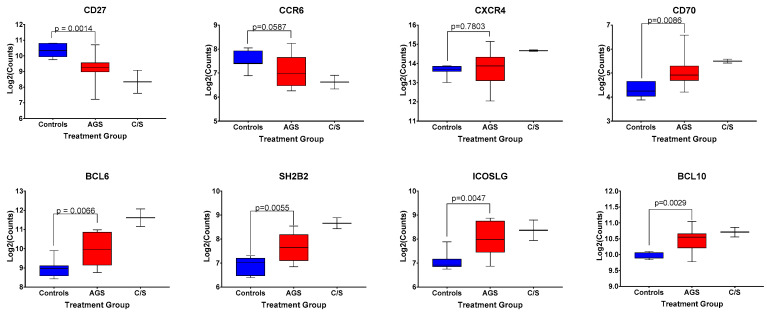
Normalized expression levels of select genes associated with B cell function among control, alpha-gal-sensitized but asymptomatic, and AGS subjects. *p* values were generated using an unpaired *t* test comparing control to AGS, with *p* < 0.05 considered statistically significant. Due to the small sample size, the sensitized but asymptomatic group was excluded from statistical analysis.

A discrete, circulating B cell phenotype enriched in individuals with alpha-gal syndrome compared to controls has been previously identified using high-dimensional mass cytometry and flow cytometry [21]. It is characterized by low levels of surface expression of CD27 and IgM, and increased expression levels of CXCR4, CCR6, CD25, and IgD [21]. Our multiplex gene transcription array did not include probes for *CD25*,* IGHD*, or *IGHM*; however, we did assess for differences in *CD27*,* CXCR4*, and *CCR6* among control, alpha-gal-sensitized, and AGS participants in our cohort (Figure 6). We found that sensitized and AGS subjects expressed lower levels of *CD27* and comparable levels of *CXCR4* transcripts compared to non-allergic controls (Figure 6, *p* = 0.0014 and p = 0.7803, respectively). There was a trend toward lower levels of expression of *CCR6* transcripts among AGS participants compared to controls (Figure 6, *p* = 0.0587). In addition, AGS and sensitized participants produced more transcripts of *CD70* (Figure 6), a cellular ligand of CD27 expressed temporarily on activated T and B cells that can regulate immunoglobulin production [22,23]. Other genes associated with B cell function that were differentially expressed between AGS and control individuals included *SH2B2*,* ICOSLG*,* BCL6* and *BCL10* (Figure 6).

## 3. Discussion

Sensitization to alpha-gal is associated with bites from ectoparasites, and in the US, the culprit ectoparasite is the lone star tick (*Amblyomma americanum*) [24]. While alpha-gal IgE increases after lone star tick bites, the mechanism by which tick bites stimulate the immune system to produce alpha-gal-specific IgE antibodies is not fully understood. In this study, we used the NanoString Pan Cancer Immune Panel, a multiplex gene transcription array that contains 770 different genes spanning 14 different immune cell types, to compare the gene expression profiles of immune genes among AGS subjects, alpha-gal-sensitized (but not allergic) subjects and non-alpha-gal allergic controls. Our goal was to assess for differences in gene expression between alpha-gal-sensitized and unsensitized individuals that might highlight molecular targets or pathways essential for the development of AGS. Nanostring technology is widely used to study cancers [25,26] and autoinflammatory and autoimmune diseases [27,28,29,30]. It has also been deployed to dissect transcription profiles in other allergic diseases, including asthma [31,32] and allergic rhinitis [33,34]. To our knowledge, this proof-of-concept study is the first application of this technology to alpha-gal mammalian meat allergy, and food allergy in general.

We found that sensitization to alpha-gal, irrespective of a history of adverse allergic reactions to ingested mammalian products, was associated with a distinct transcriptional profile in circulating peripheral blood mononuclear cells (PBMCs). Of the top 53 differentially expressed genes, 37 were upregulated in AGS participants compared to controls. They included genes involved in the NFκB signaling pathway, such as *NFKBIA*,* NFKB2*, and *REL*, as well as transcription factors such as *EGR1*, a zinc-finger and immediate early gene operational in both innate and adaptive immune cells. The differential expression of NF-κB transcription factor family members, including *NFKBIA* and *NFKB2*, has been reported in the microarray and RNA-seq-based transcriptional profiling of PBMCs from peanut-, egg- and milk-allergic children [35,36,37]. *IL1R2*, which encodes a negative regulator of pro-inflammatory signaling downstream of IL-1, associated with innate immunity and the activation of the NFκB signaling pathway [37], was also upregulated in alpha-gal-sensitized individuals (Table S3 and Figure S2). Interestingly, *IL1R2* is also overexpressed in adults with fruit and/or latex allergies [38]. In addition, it was identified as a key driver strongly linked to acute peanut allergic reactions in peanut-allergic children [37]. EGR1 regulates the expression of several genes critical for immune regulation, including *CD44*, *IL-2*, and *TNF* [39,40]. There was a significant upregulation of genes encoding MHC II molecules, such as *HLA-DRB3* and *HLA-DMA*, intimately involved in antigen presentation, as well as *CD83*, a surface receptor expressed on activated B and T cells [41] that can regulate antigen presentation.

In addition, one-sixth of the top 37 differentially expressed genes were associated with the development of type 2/Th2 immune responses and the pathogenesis of allergic diseases, with increased gene expressions of *IL13RA1*,* PLAUR*,* THBD*,* ICAM1*,* VEGF*, and *CD74. IL13RA1* encodes the IL-13Rα1 protein, which, in conjunction with IL-4Rα, forms the IL-13 receptor. IL-4 and IL-13 are powerful mediators of type-2 inflammation and can signal via the IL-13 receptor [42]. *IL13RA1* has been implicated in eosinophilic esophagitis [43] and asthma pathogenesis [44]. Levels of, and polymorphisms within, *PLAUR* (plasminogen activator, urokinase receptor) have been associated with asthma, asthma severity, and airway remodeling in asthma [45,46,47,48]. Notably, the transcriptional analysis of cultured lipid-raft-disrupted keratinocytes, whose transcriptional profiles most closely match lesional skin from atopic dermatitis (AD) patients, showed the upregulation of *PLAUR* expression [49].

*VEGF* (vascular endothelial growth factor) expression has been associated with underlying responses to an environmental allergen driving an allergic response to food (i.e., oral allergy syndrome) [50]. In grass-allergic patients sensitized to profilin, increased levels of VEGF-A protein in the oral mucosa are linked to clinically severe oral allergy syndrome [50]. Moreover, VEGF-A levels are downregulated in cow’s milk-allergic patients who undergo desensitization with oral immunotherapy (OIT) [51]. Adult patients with either cow’s milk, wheat, oat or rye allergies demonstrated increased ICAM1 protein expression in the lamina propria of the gut [52]. CD74, or the HLA invariant chain, is upregulated in T cells and eosinophils, and facilitates the trafficking of these cells to areas of high macrophage inhibitory factor (MIF) expression [53]. Atopic individuals with allergic asthma, allergic rhinitis, and atopic dermatitis frequently express high levels of MIF [53]. In a model of chemical skin injury and dermatitis, CD74 on invariant natural killer T (iNKT) cells interacted with MIF to promote iNKT cell migration to the skin [54]. The upregulation of *IL13RA1*,* PLAUR*,* ICAM1*,* VEGF*,** and *CD74* in alpha-gal-sensitized individuals seems to link genes expressed in tissues with high environmental exposures (skin, gut, airway) to the development of an IgE response. If replicated in an independent cohort of AGS individuals, this particular transcriptional signature may connect the tick bite at the skin with the development of an alpha-gal IgE response in AGS and alpha-gal-sensitized individuals.

Individuals sensitized to alpha-gal also showed increased expressions of the genes *CDKN1A*,* TNF*,* CXCL2*,* CCL3*, and *CCL3L1*. CDKN1A, also known as the p21 cell cycle inhibitor [55], and TNF have been identified as pivotal proteins in atopic dermatitis (AD) pathogenesis through a review of AD-associated protein–protein interaction networks, with some suggesting a role for these proteins in the development of itch and xerosis [56]. CXCL2, CCL3, and CCL3L1 have been associated with itch and skin pathology [57]. In our study of PBMCs, we found the upregulation of both *CCL3L1* and *CCL3* gene transcripts in alpha-gal-sensitized individuals compared to control. The increased expression of genes associated with skin pathology and itch in alpha-gal-sensitized patients may reflect the key role of the skin epithelia and skin resident lymphocytes, as well as innate immune cells, following tick bites in initiating alpha-gal sensitization.

T and B lymphocytes are critical for the sensitization phase in conventional food allergy [14], and we found that the principal component analysis and unsupervised hierarchical clustering of genes associated with T cell function and B cell function divided participants into two major clusters. For genes associated with T cell function, the majority of AGS and alpha-gal-sensitized participants clustered together, distinctly from non-sensitized, non-alpha-gal allergic controls. Specifically, 14 out of 17 alpha-gal-sensitized participants (12 with alpha-gal syndrome, and 2 sensitized but asymptomatic participants) clustered together, while just 3 AGS subjects clustered with the controls. For genes associated with B cell function, the picture is more complex. Principal component analysis showed that 15 of 17 alpha-gal-sensitized participants clustered together, and only 2 of 17 clustered with the 7 control subjects. However, unsupervised hierarchical clustering revealed that 7 of the 17 alpha-gal-sensitized subjects, or just over 40%, clustered with controls. Thus, T and B cells transcriptional states distinguished a subset of alpha-gal-sensitized individuals from unsensitized individuals. However, additional studies using larger sample sizes are clearly needed to identify a precise T or B cell transcriptional signature for alpha-gal-sensitized patients.

A subset of T cells, invariant natural killer T (iNKT) cells, can recognize immunogenic glycolipids complexed to CD1d, including alpha-gal-containing glycolipids [58]. As IgG and IgM antibodies against alpha-gal generated by human B cells depend on CD1d-mediated interactions between B cells and iNKT cells [15], there may be a role for iNKT cells and CD1d-mediated glycolipid presentation in the pathogenesis of alpha-gal allergy. We found that CD3^+^CD1d-restricted iNKT cells are rare in human peripheral blood, detected to varying degrees in both alpha-gal-sensitized and unsensitized subjects. Through flow cytometry, we found that the median frequency of circulating activated CD69^+^ iNKT cells in AGS subjects was 2.5-fold higher than in controls, although this difference did not reach statistical significance. We also examined differentially expressed genes in bulk PBMCs associated with iNKT cell development and effector function (*BCL-6*,* CD44*,* CXCR3*,* CXCR6*,* IFNG*,* TNF*,* EGR2*, and *SLAMF6*, [18]) among control, sensitized but asymptomatic, and AGS subjects. Transcripts of *CD44*, a cellular marker of antigen experience, were upregulated in alpha-gal-sensitized subjects, as was gene expression for the cytokines *IFNG* and *TNF*. Interestingly, the gene expressions of *CXCR3* and *CXCR6*, cytokine receptors important in promoting T cell migration to tissues [59], were reduced in subjects with circulating alpha-gal-specific IgE compared to controls. Polymorphisms in *CXCR3* have been associated with atopic asthma [60]. Both *CXCR3* and *CXCR6* were downregulated on T cells from birch allergic patients [59]. The downregulation of *CXCR3* and *CXCR6* gene expression in circulating mononuclear cells may be a marker of type 2, IgE-predominant responses associated with environmentally driven allergic disease.

*BCL6* transcripts were also upregulated in alpha-gal-sensitized and allergic subjects compared to controls. BCL-6 is a transcriptional regulator of MHCII-restricted T follicular helper (T_FH_) cells, germinal center B cells [20], as well as iNKT_FH_ cells, which function like MHCII-restricted T follicular helper cells, interacting with B cells that internalize lipid antigens through the B cell receptor [19]. Moreover, iNKT_FH_ can promote germinal center reactions and B cell class-switching, but do not generate long-lived antibody-producing plasma cells [19]. This is consistent with clinical experience and a recent report suggesting that alpha-gal-specific IgE decreases over time in most AGS subjects who avoid additional tick bites [61].

It is important to note that gene expression analysis was performed on bulk PBMCs, and not a purified population of iNKT cells. Moreover, all the genes associated with iNKT cell development are also expressed by conventional non-iNKT T and B lymphocytes, which are far more abundant than iNKT cells in the peripheral blood. Thus, differential expression among this gene subset in this experiment more accurately reflects differential transcriptional states in non-iNKT T and B lymphocytes. Future studies analyzing differential gene expression in a purified population of iNKT cells from control and alpha-gal-sensitized individuals are needed to determine whether sensitization to alpha-gal promotes transcriptional differences in iNKT cells.

Cox and colleagues have described a discrete, circulating B cell phenotype enriched in individuals with alpha-gal syndrome compared to controls, using flow cytometry and high-dimensional mass cytometry, characterized by low levels of surface expression of CD27 and IgM, and increased expression levels of CXCR4, CCR6, CD25, and IgD [21]. In our cohort, we found that sensitized and AGS subjects expressed lower levels of *CD27* transcripts compared to non-allergic controls, with mRNA expression levels mirroring protein expression in the B cell subset described by Cox et al. [21]. However, differences in *CXCR4* expression and *CCR6* expression in alpha-gal-sensitized and AGS participants compared to controls did not reach statistical significance in this cohort. This contrasts with the increased CXCR4 and CCR6 protein expression observed by Cox et al. in the AGS B cell subset [21]. Comparisons of CCR6 mRNA and protein expression in other experimental systems seem to find concordance between CCR6 transcript and CCR6 protein expression [62,63]. The reduction in *CCR6* gene expression that we observed may reflect the fact that we examined *CCR6* expression across PBMCs, and not strictly within the circulating B cell population. In addition, compared to controls, there seemed to be an induction of *CD70* gene expression in AGS and sensitized participants. This is particularly notable since CD70 is a cellular ligand of CD27 expressed temporarily on activated T and B cells, which regulates IgG production [22] and can enhance IgE synthesis by B cells [23].

There are several limitations to this study, including the small number of study subjects, in particular control subjects, and alpha-gal-sensitized but asymptomatic subjects. There were also statistically significant differences in age and sex distribution among the AGS, sensitized, and control groups. The self-reported racial and ethnic identity of our alpha-gal cohort is predominantly white. The differentially expressed genes identified in this study need to be confirmed in larger, more diverse cohorts, better matched for age and sex distribution. Future studies should also explore whether the observations noted in this study are replicated in cohorts of alpha-gal-allergic patients identified in South Africa, Japan, Australia and Europe. In addition, we examined PBMCs in bulk rather than specific cell types (like B or T cells specifically). Using this approach, we may have missed differentially expressed gene targets within specific cell populations because the signal is diluted within the heterogeneous cell population studied. In addition, gene expression in circulating PBMCs in alpha-gal-sensitized individuals may differ substantially from gene expression in the tissues at the site of a tick bite.

Despite its limitations, this study highlights pathways, cell subsets, and gene targets to explore further in the quest to understand how sensitization occurs in alpha-gal syndrome.

## 4. Materials and Methods

### 4.1. Patient and Sample Collection

#### 4.1.1. Subjects

We recruited subjects, ≥17 years of age, with and without alpha-gal mammalian meat allergy following approval from the University of North Carolina—Chapel Hill Institutional Review Board (UNC IRB #16-1533, initial approval date: 1 November 2016; renewal: 24 July 2020). We obtained informed consent from all subjects for the use of their biological specimens in the experiments described. Subjects with alpha-gal allergy were defined as having allergic symptoms after ingesting mammalian meat and serum IgE antibodies to alpha-gal (Table S1). Symptomatic subjects reported allergic symptoms ≥3 h after meat ingestion. Control subjects were negative for serum alpha-gal-specific (s)IgE (<0.1 IU/mL). “Control/Sensitized” (C/S) subjects possessed detectable circulating alpha-gal-specific IgE antibodies, but reported no allergic symptoms following ingestion of alpha-gal.

We recruited 40 total participants for this study (Tables S1 and S2). Of these, 21 provided PBMC samples for the multiplex gene expression analysis only; 16 provided PBMCs for flow cytometry only, and 3 participants provided PBMC samples for both analyses. For the NanoString immune gene expression profiling there were 15 subjects with alpha-gal syndrome (AGS), 7 controls, and 2 control/sensitized subjects. For the invariant natural killer T (iNKT) cell flow cytometry studies, there were 11 AGS subjects and 8 controls. All 19 participants in the iNKT cell flow cytometry study had aliquots of PBMCs stained with human CD1d tetramers loaded with PBS57, an alpha-galactosylceramide analog (See “4.4. Cell surface staining, tetramer staining, and flow cytometry” for additional details).

The exclusion criteria included the following: (1) subjects with complex medical problems or chronic health problems affecting major organ systems that would be placed at increased risk from blood draws; (2) subjects who received chronic immunosuppressive therapy (i.e., azathioprine and cyclosporine A) within 6 months of the blood draw; (3) subjects with a history of daily oral prednisone use within 1 month prior to blood draw; (4) subjects with known anemia, defined as hematocrit < 30 mg/dL; (5) subjects with evidence of active infection, including fever (defined as T ≥ 38.3 °C), white blood cell count ≤ 4000 or ≥ 12,000/μL, or positive blood cultures at the time of evaluation; (6) subjects unable to provide informed consent.

#### 4.1.2. Collecting Blood for PBMC Isolation and Plasma Collection

Venous blood was drawn into acid citrate dextrose (ACD) solution (Sigma-Aldrich, St. Louis, MO, USA). Whole blood was centrifuged and plasma collected. Plasma samples were stored at 4 °C until analysis.

### 4.2. Messenger RNA (mRNA) Isolation from Peripheral Blood Mononuclear Cells

Peripheral blood mononuclear cells (PBMCs) were isolated from whole blood taken from study subjects using a Ficoll–Paque gradient (GE Healthcare Bio-Sciences, Pittsburgh, PA, USA). PBMCs were resuspended at 5 × 10^6^ cells/mL in freezing media (50% fetal calf serum (FCS)/20% dimethyl sulfoxide (DMSO)/30% RPMI 1640 media) and stored at −80 °C until use. Total RNA was extracted using a Qiagen RNeasy (Qiagen, Inc., Toronto, Canada) following the manufacturer’s instructions. RNA concentration was quantitated by measuring the absorbance at 260 nm using a NanoDrop ND-100 spectrophotometer (NanoDrop Technologies, Wilmington, DE, USA) and an A260/A280 ratio of 1.9–2.1 was considered as pure RNA. Further RNA integrity was determined by an Agilent 2200 TapeStation system (Agilent Technologies, Inc., Santa Clara, CA, USA), and RNA with an RNA integrity number equivalent (RINe) of 8 or more was used for NanoString gene expression analysis.

### 4.3. NanoString PanCancer Immune Profile Panel Multiplex Gene Expression Profiling

We used the digital multiplexed NanoString^®^ nCounter analysis system (NanoString Technologies, Seattle, WA, USA) to perform gene expression profiling with 200 ng total RNA from each subject sample as input. We analyzed our RNA samples using the nCounter PanCancer immune profile panel, which incorporated 770 human genes—40 reference (housekeeping genes) and 730 immune genes (NanoString Technologies). Raw data (Table S5) were normalized in NanoString nSolver 4.0 software against 6 positive and 8 negative controls to accommodate background noise and sample variation associated with the NanoString platform (Table S6). Then, the nSolver 4.0 software performed reference gene normalization using the GeNorm Algorithm with 40 housekeeping genes. Differentially expressed genes from PBMCs were determined using the Benjamini–Hochberg adjusted *t* test, with significance set at *p* < 0.05. The database for annotation, visualization, and integrated discovery (DAVID) was used for functional gene annotation and pathway analysis, and included genes expressing >1 log_2_ fold change with significant pathway enrichment accepted at *p* < 0.05 and a false discovery rate of *p* < 0.05.

Data were also analyzed by ROSALIND^®^ (https://rosalind.onramp.bio/, accessed 7 March 2021), with a HyperScale architecture developed by ROSALIND, Inc. (San Diego, CA, USA). Read distribution percentages, violin plots, identity heatmaps, and sample multidimensional scaling (MDS) plots were generated as part of the QC step. Normalization, fold changes and *p*-values were calculated using criteria provided by NanoString, as described above. ROSALIND^®^ follows the nCounter^®^ Advanced Analysis protocol of dividing counts within a lane by the geometric mean of the normalizer probes from the same lane. Housekeeping probes to be used for normalization were selected based on the geNorm algorithm as implemented in the NormqPCR R library^1^. The abundance of various cell populations was calculated on ROSALIND using the NanoString Cell Type Profiling Module. ROSALIND performed a filtering of the cell-type profiling results to include results that had scores with a *p*-value greater than or equal to 0.05. Fold changes and *p*-values were calculated using the optimal method as described in the nCounter^®^ Advanced Analysis 2.0 User Manual. P-value adjustment was performed using the Benjamini–Hochberg method of estimating false discovery rates (FDR). For the differentially expressed genes identified with the filter of adjusted *p*-value ≤ 0.02, the UniProt Knowledgebase (UniProtKB) database and the nCounter PanCancer immune profile panel annotation database were referenced to generate an “Immune Response Category.”

Group differences in immune gene expression were compared with Graphpad Prism Software v7.03 (San Diego, CA, USA), using unpaired t-tests with statistical significance set at *p* < 0.05.

### 4.4. Cell Surface Staining, Tetramer Staining, and Flow Cytometry

Anti-CD3-FITC (clone UCHT1) and anti-CD69-APC (clone FN50) were both purchased from BD Biosciences (San Jose, CA, USA). Unloaded human CD1d tetramers, and CD1d tetramers loaded with the alpha-galactosylceramide analog PBS57 [17] and complexed with PE-conjugated streptavidin, were provided by the National Institute of Allergy and Infectious Diseases tetramer facility (Emory University Vaccine Center, Atlanta, GA, USA). PBMCs were isolated from the whole blood of control and AGS subjects using a Ficoll–Paque gradient and stained with fluorescently labeled antibodies against CD3 and CD69 in flow cytometry staining buffer (phosphate buffered saline, 2% fetal calf serum, 0.02% NaN_3_). Before fixation and after monoclonal antibody staining, cells were stained with tetramer for 30 min at 4 °C, as described by Kamath et al. [64]. Samples were acquired on a CyAN ADP flow cytometer (Beckman Coulter, Brea, CA, USA) or on an Attune NxT flow cytometer (Thermo Fisher Scientific, Waltham, MA, USA) and analyzed using FlowJo v10 software (FlowJo LLC, Ashland, OR, USA).

## Data Availability

Data supporting reported results can be found in Supplementary Materials. Table S5 (“Raw Transcript Counts”) lists all the raw gene transcript counts for every gene included on the NanoString nCounter PanCancer immune profile panel for each sample. Table S6 (“Normalized Transcript Counts”) lists the normalized counts for each sample.

## Supplementary Materials

The following are available online at https://www.mdpi.com/1422-0067/22/6/3185/s1.

## Abbreviations

AD	Atopic dermatitis
AGS	Alpha-gal syndrome
DEG	Differentially expressed genes
ICAM1	Intracellular adhesion molecule 1
iNKT	Invariant natural killer T cell
PBMCs	Peripheral blood mononuclear cells
PLAUR	Plasminogen activator, urokinase receptor
