# Supplementary material for: T and B Lymphocyte Transcriptional States Differentiate between Sensitized and Unsensitized Individuals in Alpha-Gal Syndrome
Source: Int J Mol Sci. 2021 Mar 20;22(6):3185. doi: 10.3390/ijms22063185 (PMC8003943; doi:10.3390/ijms22063185)
Supplement: Supplementary file 1 [file ijms-22-03185-s001.zip › SUP/ijms-1101923-GEpdf.pdf]

Figure Legends

Figure S1. Differentially expressed genes in PBMCs from controls and alpha-gal sensitized but asymptomatic participants. Volcano plots show –log10(p-value) and log 2(fold change) in gene expression for sensitized but asymptomatic ("C.S.") participants compared to control. Highly statistically significant genes are at the top of the plot above the horizontal lines which represent p-value thresholds adjusted using the Benjamini-Hochberg method of estimating false discovery rates (FDR). Highly differentially expressed genes fall to the left and right. There are 80 genes where adjusted p-value for differential gene expression is $p<0.05$ and 45 genes where adjusted p-value is $p<0.02$. The 40 most statistically significant genes are labeled in the plot.

Figure S2. Differentially expressed genes common to both AGS and alpha-gal sensitized but asymptomatic subjects compared to controls. Panel (A) shows the 53 differentially expressed genes (DEG) with adjusted p-value <0.02 in AGS vs. Control. Thirty-seven genes were upregulated (green) and 16 genes were downregulated (purple) in AGS compared to control. Panel (B) shows the 45 genes with adjusted p-value <0.02 in sensitized but asymptomatic (C/S) compared to control. Thirty-one genes were upregulated (green) and 14 downregulated (purple). The 21 upregulated genes common to AGS and C/S vs. Controls are boxed in red; the 7 common downregulated genes are boxed in blue. (C) Venn diagram depicting differentially expressed genes common to both AGS and C/S when each group is compared to controls. Red represents upregulation and blue, downregulation.

Figure S3. Cell type profiling across all samples. Adaptive (B and T) lymphocyte populations (circled in red) are abundant in PBMC preparations from study participants. Nanostring nSolver Software uses pre-designated immune cell type transcriptional signatures within the nCounter expression panel to quantify the relative abundance of the 11 different immune cell types listed across the X-axis in the figure. In this barplot of p-values across cell types, p-values are –log10 transformed. Bars above the solid black line indicate statistically significant cell types at an alpha level of 0.01. Bars above the dashed line indicate statistically significant cell types at an alpha level of 0.001.

Figure S4. Circulating, activated CD1d-restricted iNKT cells are detectable in both control and alpha-gal allergic subjects. (A) Representative flow cytometry plots and (B) Summary graphs of $CD3^{+}hCD1dtetramer^{+}$iNKT cell frequency (C) $CD3^{+}$ hCD1dtetramer negative non-iNKT T cell frequency (D) Frequency of $CD69^{+}$ activated cells among iNKT cells and (E) Frequency of $CD69^{+}$ activated cells among $CD3^{+}$non-iNKT T cells detected in PBMCs from control and alpha-gal allergic subjects. Invariant natural killer T cells were identified using anti-CD3 and human CD1d tetramers loaded with the alpha-galactosylceramide analog PBS57 ("hCD1dtetramer"). p-values generated using the Mann-Whitney test with $p<0.05$ considered significant. AGS = Alpha-gal syndrome.

Figure 1. Study Participant Characteristics for Multiplex Gene Expression Analysis Cohort. (A) Age. (B) Sex. (C) Self-reported race. (D) Symptom distribution. (E) Serum alpha-gal-specific IgE. (F) Serum total IgE. Control (n=7); Sensitized but asymptomatic ("Control/Sens," n=2); Alpha-gal syndrome ("AGS" n=15). P values were generated using an unpaired t test comparing AGS to control, with $p<0.05$ considered significant. Due to the small sample size, the sensitized but asymptomatic group was excluded from statistical analysis**.**

Figure 2. Differentially expressed genes in PBMCs from alpha-gal allergic participants compared to controls. (A) Volcano plots show –log10(p-value) and Log 2 fold change in gene expression for AGS

participants compared to control. Highly statistically significant genes are at the top of the plot above the horizontal lines which represent p-value thresholds adjusted using the Benjamini-Hochberg method of estimating false discovery rates (FDR). Highly differentially expressed genes fall to the left and right. There are 81 genes where adjusted p-value for differential gene expression is $p<0.05$ and 53 genes where adjusted p-value is $p<0.02$. The 40 most statistically significant genes are labeled in the plot. (B) Graphic lists the 53 genes with adjusted p-value <0.02. Thirty-seven gene transcripts highlighted in green were upregulated and 16 gene transcripts highlighted in purple were downregulated in AGS compared to control.

Figure 3. Normalized expression levels of selected immune response-related genes upregulated in PBMCs from AGS subjects compared to controls. (A) Genes involved in transcriptional regulation and/or the NFkappaB pathway. (B) Genes associated with antigen presentation and MHC II surface expression. (C) Genes associated with type 2 / Th2 allergic immune responses. (D) Genes associated with itch and allergic dermatitis. P-values were generated using an unpaired t test comparing AGS to controls, with $p<0.05$ considered significant. Due to the small sample size, the sensitized but asymptomatic (“C/S”) group was excluded from statistical analysis. AGS = alpha-gal syndrome.

Figure 4. Transcriptional immune profiles for B and T cell function from control and alpha-gal sensitized subjects are largely distinct. Heatmap of the normalized gene expression data and unsupervised hierarchical clustering of genes associated with (A) T cell function and (B) B-cell function. (C) Principal component analysis of gene expression data associated with T cell function and (D) B cell function. Control (n=7); Sensitized (“CS” n=2); Alpha-gal syndrome (“AGS” n=15).

Figure 5. Normalized expression levels of select genes associated with iNKT cell development and effector function in bulk PBMCs from control, alpha-gal sensitized but asymptomatic, and AGS subjects. P-values were generated using an unpaired t test comparing AGS to controls, with $p<0.05$ considered statistically significant. Due to the small sample size, the sensitized but asymptomatic group was excluded from statistical analysis.

Figure 6. Normalized expression levels of select genes associated with B cell function among control, alpha-gal sensitized but asymptomatic, and AGS subjects. P-values were generated using an unpaired t test comparing control to AGS, with $p<0.05$ considered statistically significant. Due to the small sample size, the sensitized but asymptomatic group was excluded from statistical analysis.

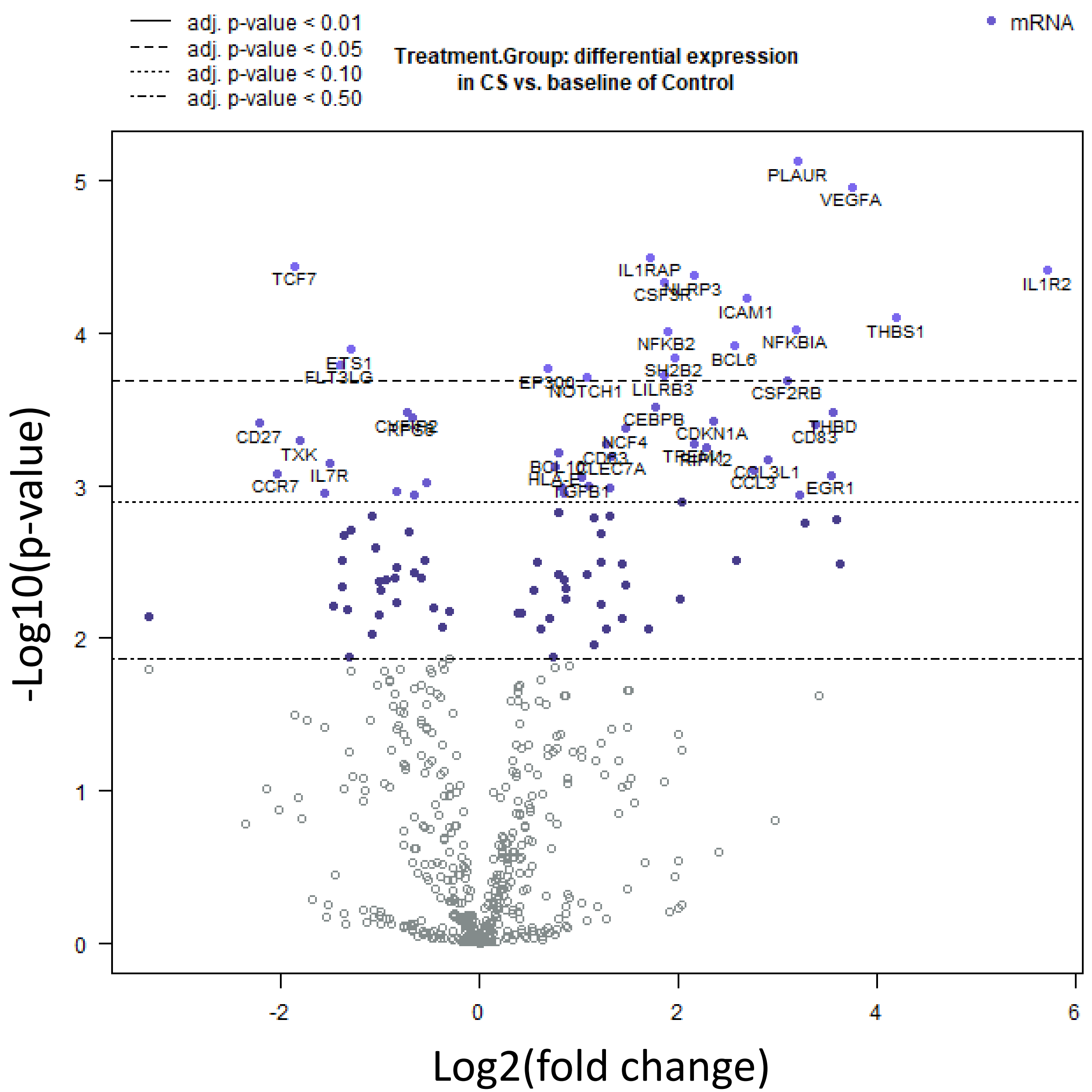
adj. p-value < 0.01
adj. p-value < 0.05
adj. p-value < 0.10
adj. p-value < 0.50
Treatment.Group: differential expression
in CS vs. baseline of Control
mRNA
-Log10(p-value)
Log2(fold change)
PLAUR
VEGFA
TCF7
IL1RAP
NLRP3
IL1R2
ICAM1
THBS1
NFKB2
NFKBIA
BCL6
ETS1
FLT3LG
SH2B2
EP300
NOTCH1
LILRB3
CSF2RB
CEBPB
CD27
CDKN1A
THBD
CD83
TXK
NCF4
IL7R
CLEC7A
CCL3L1
CCR7
CCL3
EGR1
TGFB1

Figure S1.

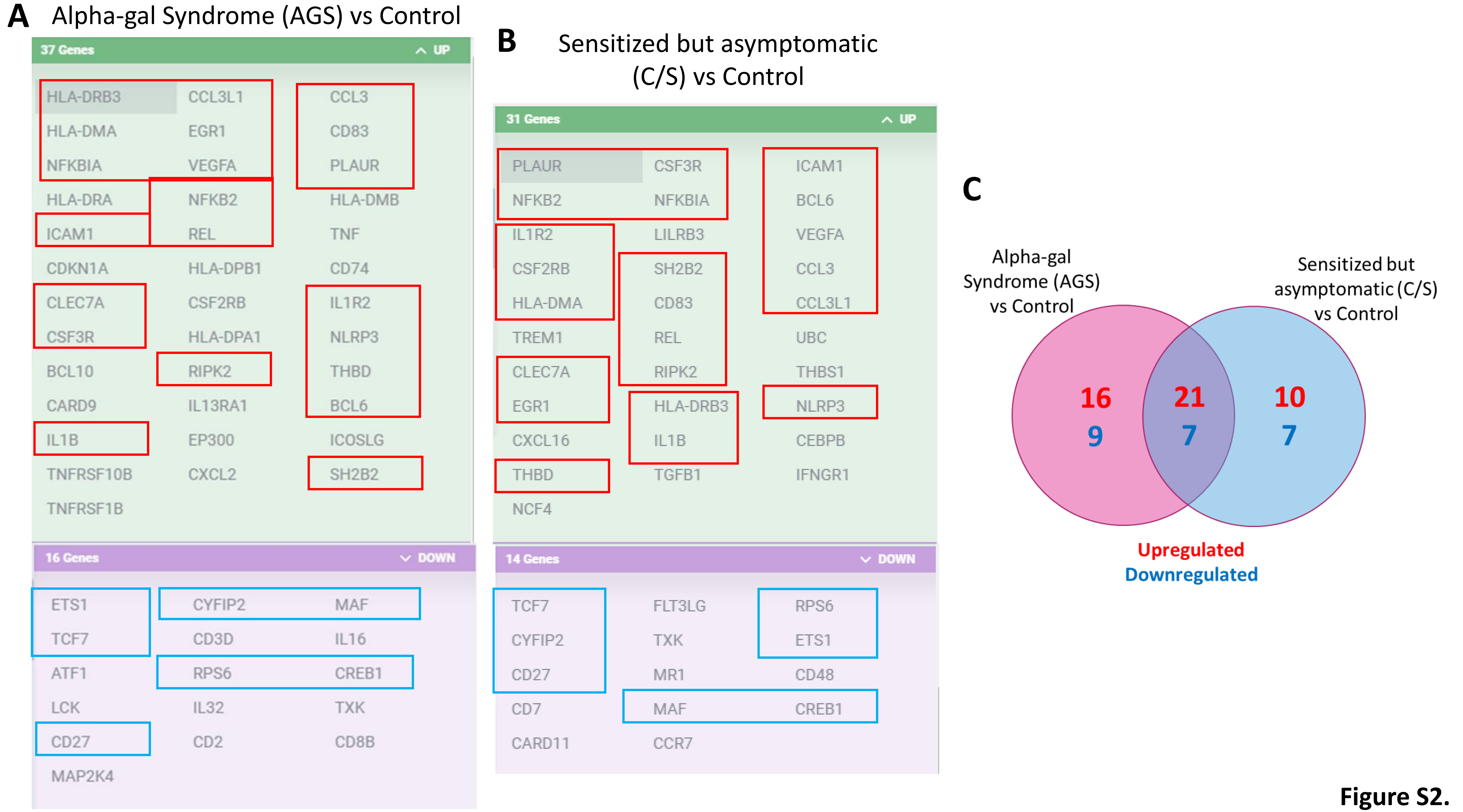

A
Alpha-gal Syndrome (AGS) vs Control
37 Genes
UP
HLA-DRB3
CCL3L1
CCL3
HLA-DMA
EGR1
CD83
NFKBIA
VEGFA
PLAUR
HLA-DRA
NFKB2
HLA-DMB
ICAM1
REL
TNF
CDKN1A
HLA-DPB1
CD74
CLEC7A
CSF2RB
IL1R2
CSF3R
HLA-DPA1
NLRP3
BCL10
RIPK2
THBD
CARD9
IL13RA1
BCL6
IL1B
EP300
ICOSLG
TNFRSF10B
CXCL2
SH2B2
TNFRSF1B
16 Genes
DOWN
ETS1
CYFIP2
MAF
TCF7
CD3D
IL16
ATF1
RPS6
CREB1
LCK
IL32
TXK
CD27
CD2
CD8B
MAP2K4
B
Sensitized but asymptomatic (C/S) vs Control
31 Genes
UP
PLAUR
CSF3R
ICAM1
NFKB2
NFKBIA
BCL6
IL1R2
LILRB3
VEGFA
CSF2RB
SH2B2
CCL3
HLA-DMA
CD83
CCL3L1
TREM1
REL
UBC
CLEC7A
RIPK2
THBS1
EGR1
HLA-DRB3
NLRP3
CXCL16
IL1B
CEBPB
THBD
TGFB1
IFNGR1
NCF4
14 Genes
DOWN
TCF7
FLT3LG
RPS6
CYFIP2
TXK
ETS1
CD27
MR1
CD48
CD7
MAF
CREB1
CARD11
CCR7
C
Alpha-gal Syndrome (AGS) vs Control
Sensitized but asymptomatic (C/S) vs Control
16
9
21
7
10
7
Upregulated
Downregulated

Figure S2.

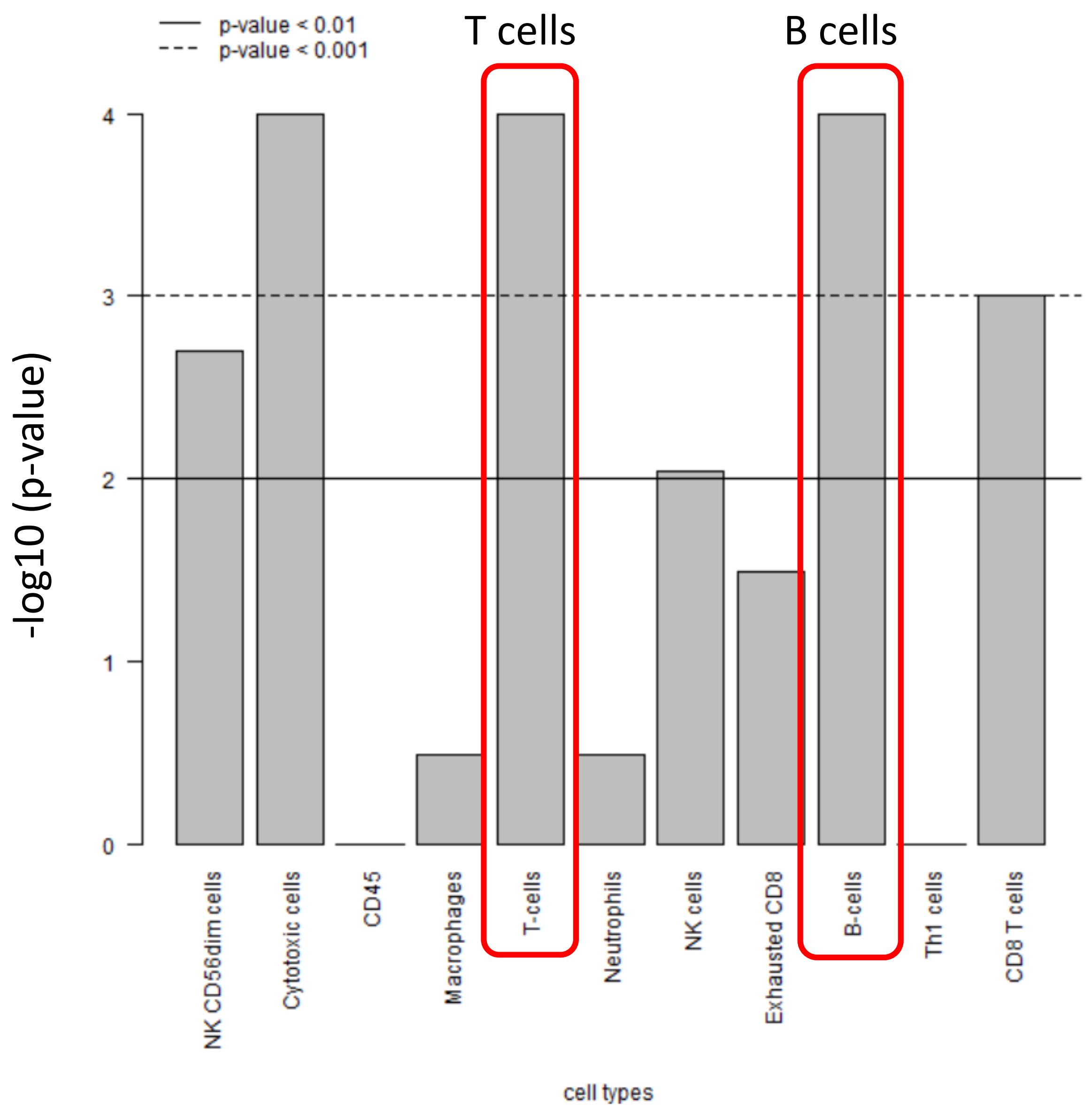
p-value < 0.01
p-value < 0.001
T cells
B cells
-log10 (p-value)
4
3
2
1
0
NK CD56dim cells
Cytotoxic cells
CD45
Macrophages
T-cells
Neutrophils
NK cells
Exhausted CD8
B-cells
Th1 cells
CD8 T cells
cell types

Figure S3.

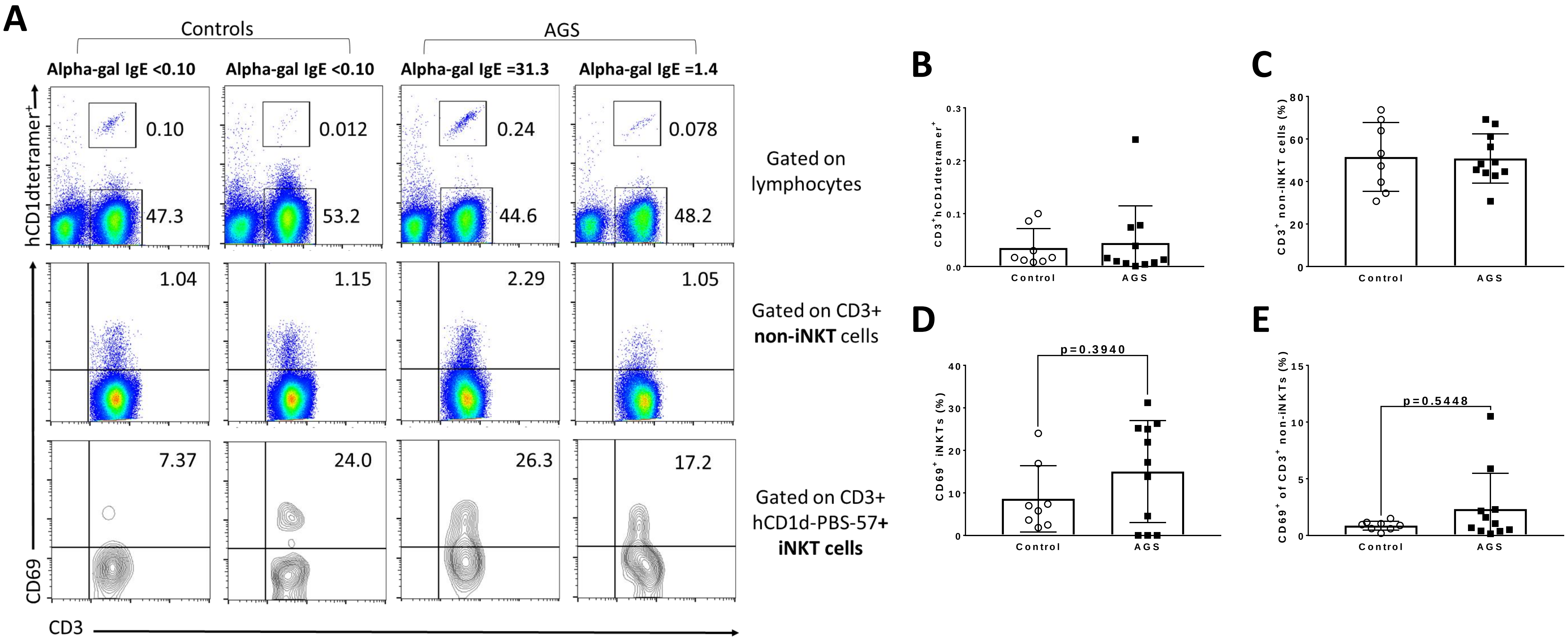
A
Controls
AGS
Alpha-gal IgE <0.10
Alpha-gal IgE <0.10
Alpha-gal IgE =31.3
Alpha-gal IgE =1.4
hCD1dtetramer+
0.10
47.3
0.012
53.2
0.24
44.6
0.078
48.2
Gated on lymphocytes
1.04
1.15
2.29
1.05
Gated on CD3+ non-iNKT cells
7.37
24.0
26.3
17.2
Gated on CD3+ hCD1d-PBS-57+ iNKT cells
CD69
CD3
B
CD3+hCD1dtetramer+
Control
AGS
C
CD3+ non-iNKT cells (%)
Control
AGS
D
p=0.3940
CD69+ iNKTs (%)
Control
AGS
E
p=0.5448
CD69+ of CD3+ non-iNKTs (%)
Control
AGS

Figure S4.

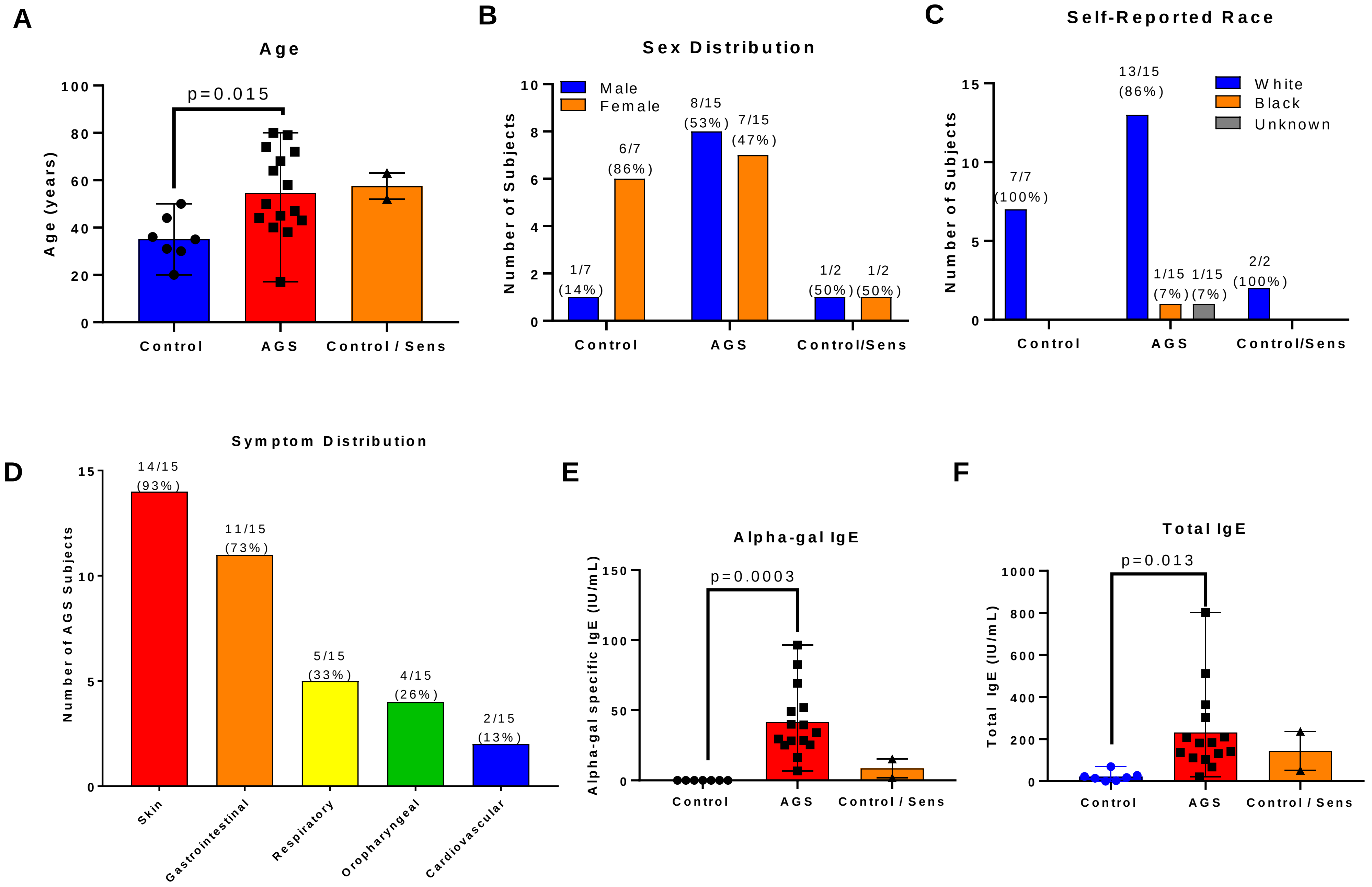
A
Age
p=0.015
Age (years)
100
80
60
40
20
0
Control
AGS
Control / Sens
B
Sex Distribution
Male
Female
Number of Subjects
1/7 (14%)
6/7 (86%)
8/15 (53%)
7/15 (47%)
1/2 (50%)
1/2 (50%)
Control
AGS
Control/Sens
C
Self-Reported Race
White
Black
Unknown
Number of Subjects
7/7 (100%)
13/15 (86%)
1/15 (7%)
1/15 (7%)
2/2 (100%)
Control
AGS
Control/Sens
D
Symptom Distribution
Number of AGS Subjects
14/15 (93%)
11/15 (73%)
5/15 (33%)
4/15 (26%)
2/15 (13%)
Skin
Gastrointestinal
Respiratory
Oropharyngeal
Cardiovascular
E
Alpha-gal IgE
p=0.0003
Alpha-gal specific IgE (IU/mL)
150
100
50
0
Control
AGS
Control / Sens
F
Total IgE
p=0.013
Total IgE (IU/mL)
1000
800
600
400
200
0
Control
AGS
Control / Sens

Figure 1.

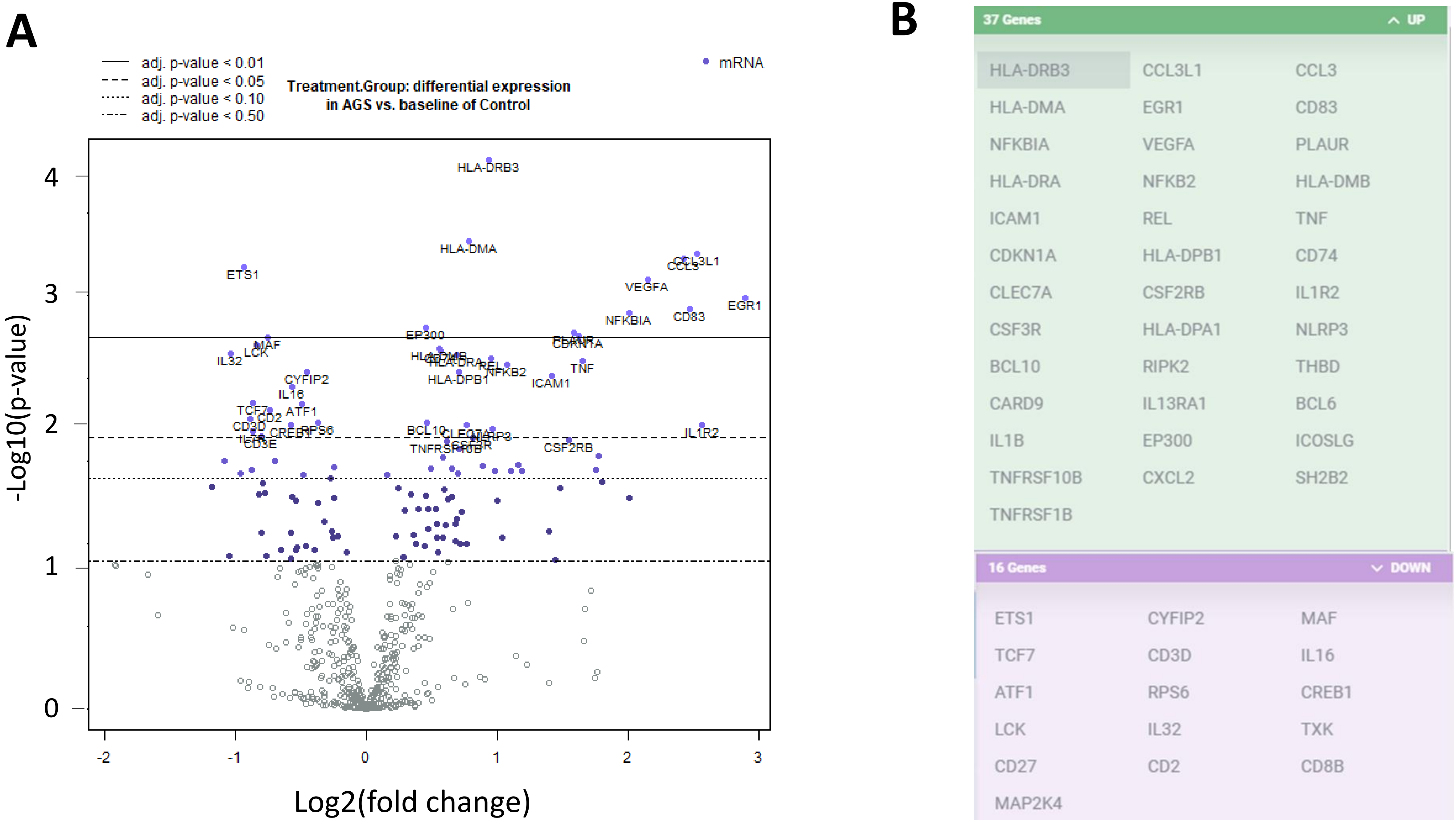
A
adj. p-value < 0.01
adj. p-value < 0.05
adj. p-value < 0.10
adj. p-value < 0.50
Treatment.Group: differential expression
in AGS vs. baseline of Control
mRNA
-Log10(p-value)
Log2(fold change)
B
37 Genes
UP
HLA-DRB3
CCL3L1
CCL3
HLA-DMA
EGR1
CD83
NFKBIA
VEGFA
PLAUR
HLA-DRA
NFKB2
HLA-DMB
ICAM1
REL
TNF
CDKN1A
HLA-DPB1
CD74
CLEC7A
CSF2RB
IL1R2
CSF3R
HLA-DPA1
NLRP3
BCL10
RIPK2
THBD
CARD9
IL13RA1
BCL6
IL1B
EP300
ICOSLG
TNFRSF10B
CXCL2
SH2B2
TNFRSF1B
16 Genes
DOWN
ETS1
CYFIP2
MAF
TCF7
CD3D
IL16
ATF1
RPS6
CREB1
LCK
IL32
TXK
CD27
CD2
CD8B
MAP2K4

Figure 2.

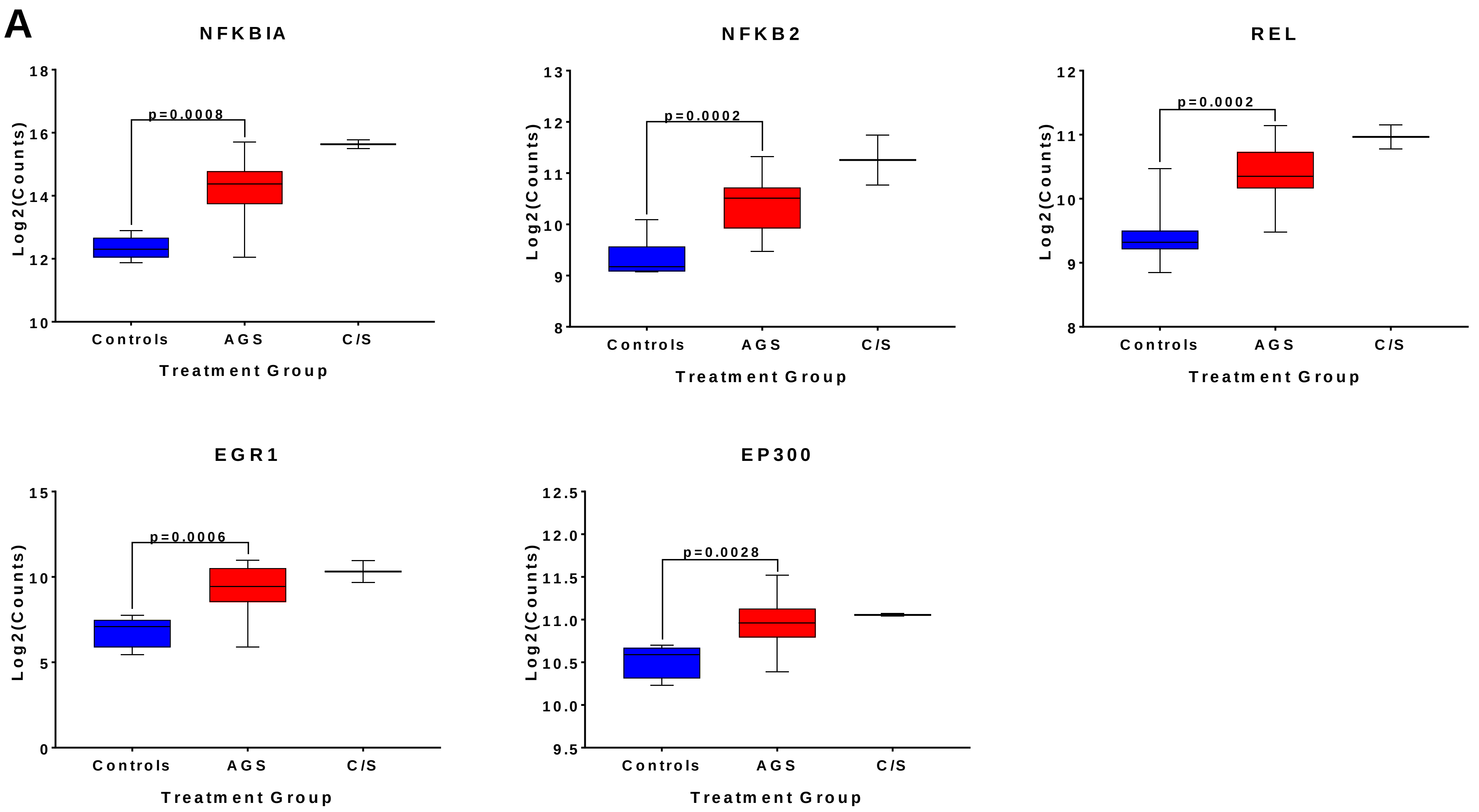
A
NFKBIA
18
16
14
12
10
Log2(Counts)
p=0.0008
Controls
AGS
C/S
Treatment Group
NFKB2
13
12
11
10
9
8
Log2(Counts)
p=0.0002
Controls
AGS
C/S
Treatment Group
REL
12
11
10
9
8
Log2(Counts)
p=0.0002
Controls
AGS
C/S
Treatment Group
EGR1
15
10
5
0
Log2(Counts)
p=0.0006
Controls
AGS
C/S
Treatment Group
EP300
12.5
12.0
11.5
11.0
10.5
10.0
9.5
Log2(Counts)
p=0.0028
Controls
AGS
C/S
Treatment Group

Figure 3A.

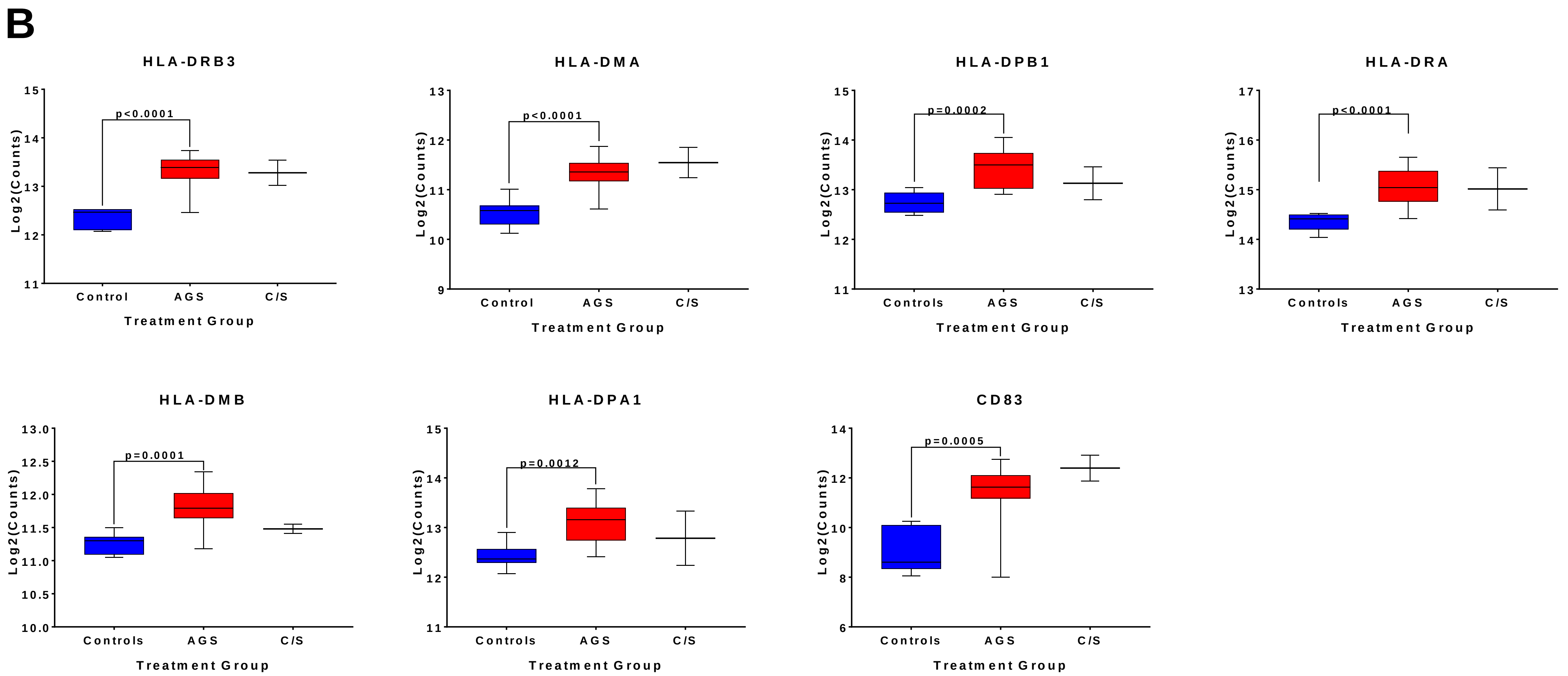
B
HLA-DRB3
p<0.0001
Log2(Counts)
Control
AGS
C/S
Treatment Group
HLA-DMA
p<0.0001
HLA-DPB1
p=0.0002
Controls
HLA-DRA
p<0.0001
HLA-DMB
p=0.0001
HLA-DPA1
p=0.0012
CD83
p=0.0005

Figure 3B.

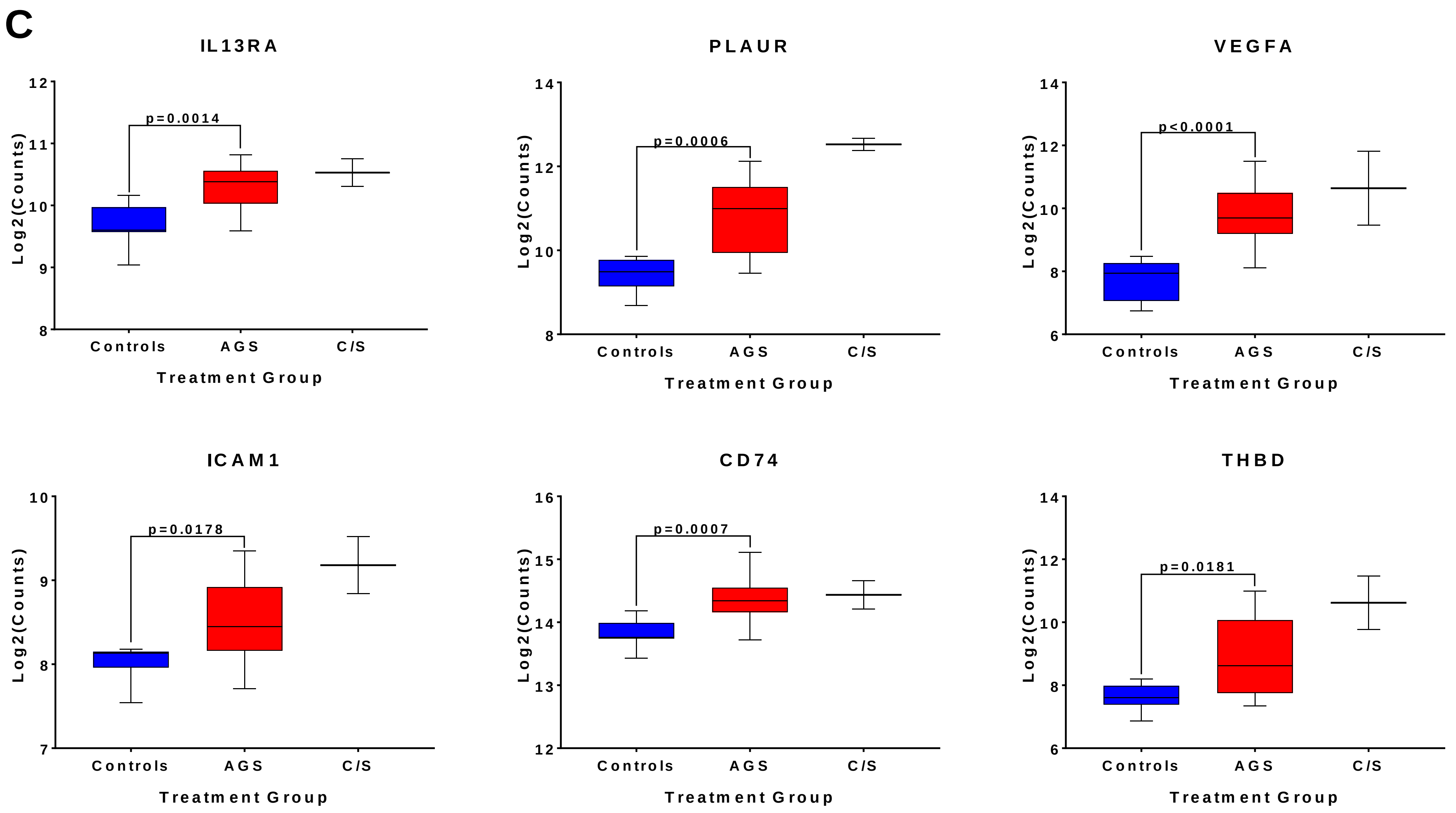
C
IL13RA
p=0.0014
PLAUR
p=0.0006
VEGFA
p<0.0001
ICAM1
p=0.0178
CD74
p=0.0007
THBD
p=0.0181
Log2(Counts)
Controls
AGS
C/S
Treatment Group

Figure 3C.

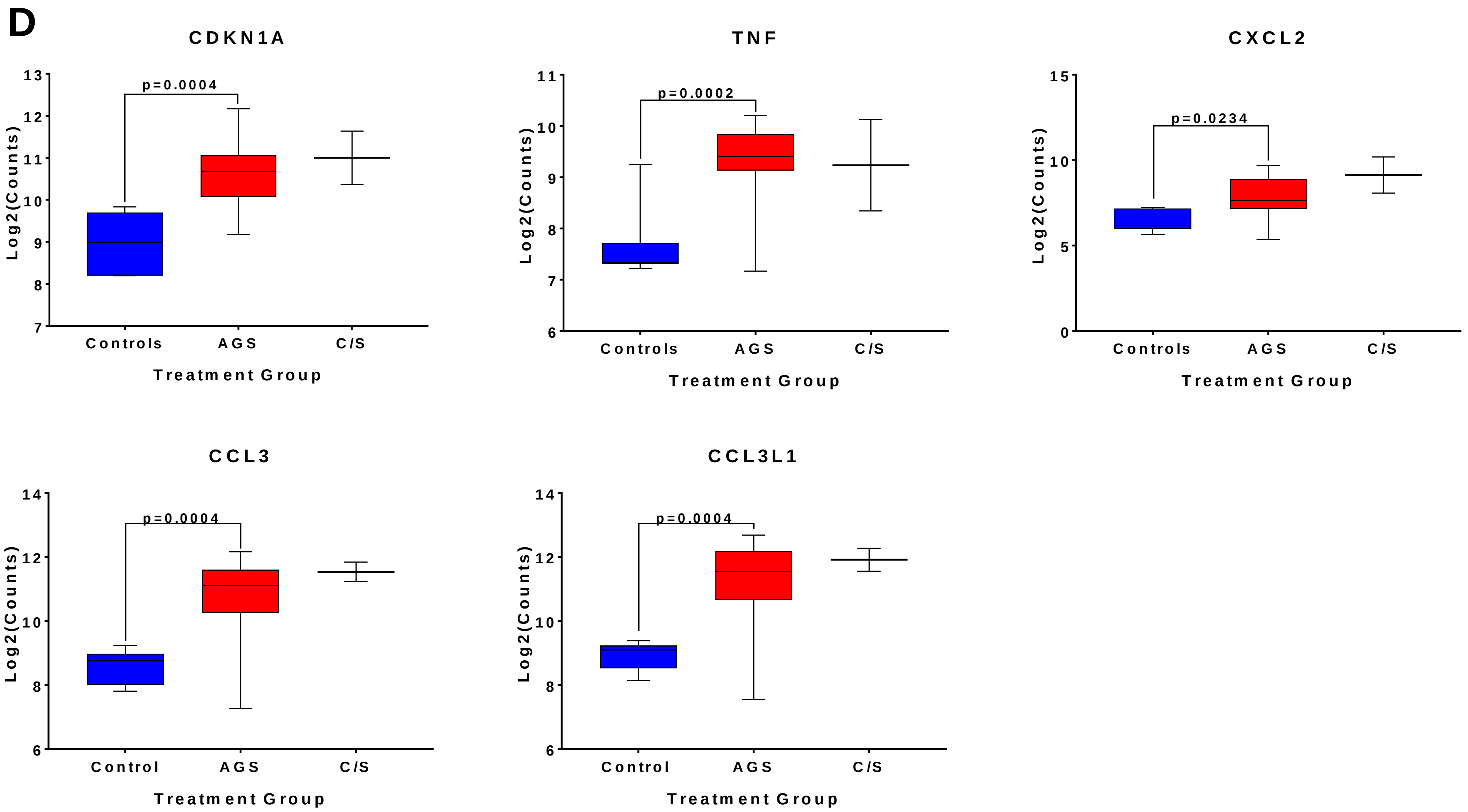
D
CDKN1A
p=0.0004
Log2(Counts)
13
12
11
10
9
8
7
Controls
AGS
C/S
Treatment Group
TNF
p=0.0002
Log2(Counts)
11
10
9
8
7
6
Controls
AGS
C/S
Treatment Group
CXCL2
p=0.0234
Log2(Counts)
15
10
5
0
Controls
AGS
C/S
Treatment Group
CCL3
p=0.0004
Log2(Counts)
14
12
10
8
6
Control
AGS
C/S
Treatment Group
CCL3L1
p=0.0004
Log2(Counts)
14
12
10
8
6
Control
AGS
C/S
Treatment Group

Figure 3D.

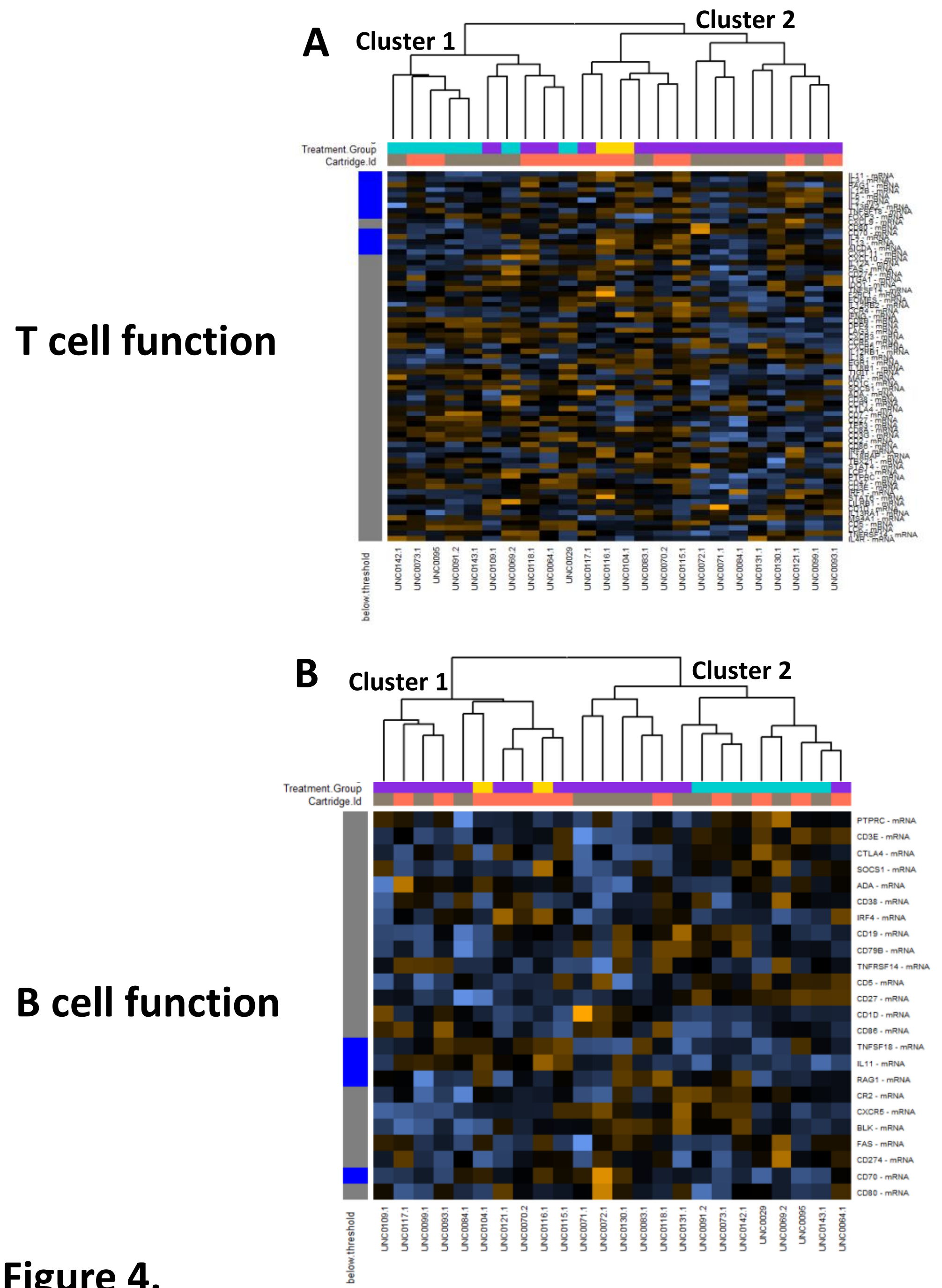

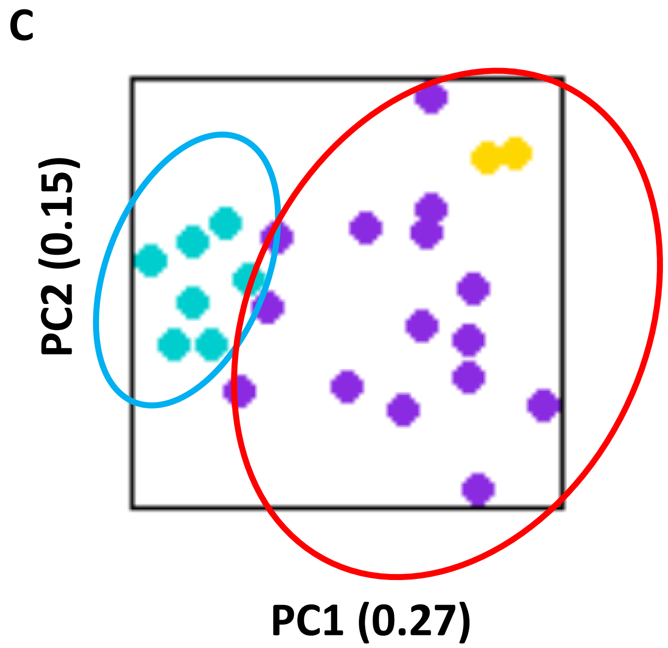

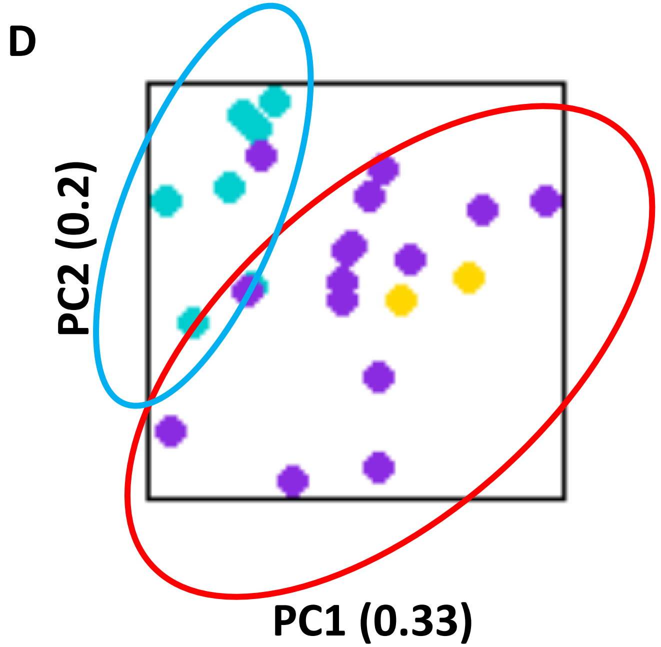

Figure 4. Transcriptional immune profiles for B and T cell function from control and alpha-gal sensitized subjects are largely distinct. Heatmap of the normalized gene expression data and unsupervised hierarchical clustering of genes associated with (A) T cell function and (B) B-cell function. (C) Principal component analysis of gene expression data associated with T cell function gene and (D) B cell function. Control (n=7); Sensitized ("C/S," n=2); Alpha-gal syndrome ("AGS" n=15)

Figure 4.

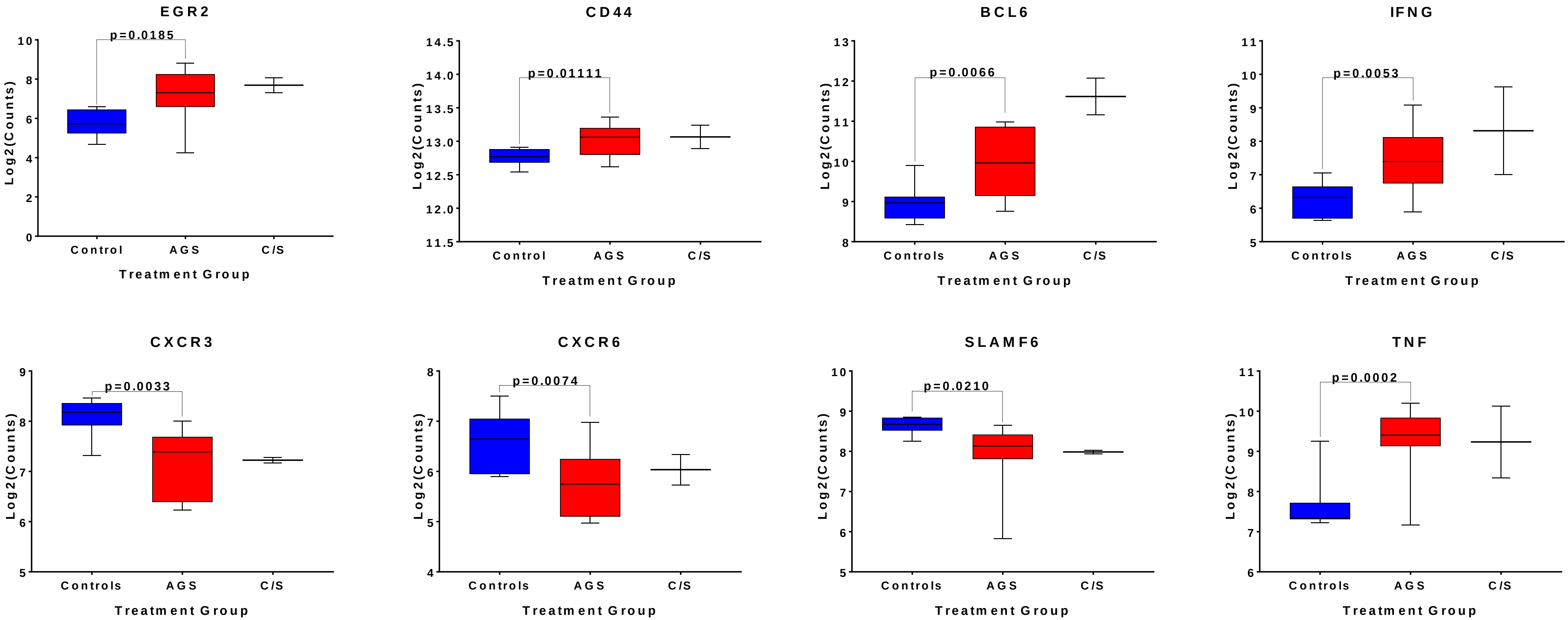
EGR2
p=0.0185
Log2(Counts)
Control
AGS
C/S
Treatment Group
CD44
p=0.01111
BCL6
p=0.0066
Controls
IFNG
p=0.0053
CXCR3
p=0.0033
CXCR6
p=0.0074
SLAMF6
p=0.0210
TNF
p=0.0002

Figure 5.

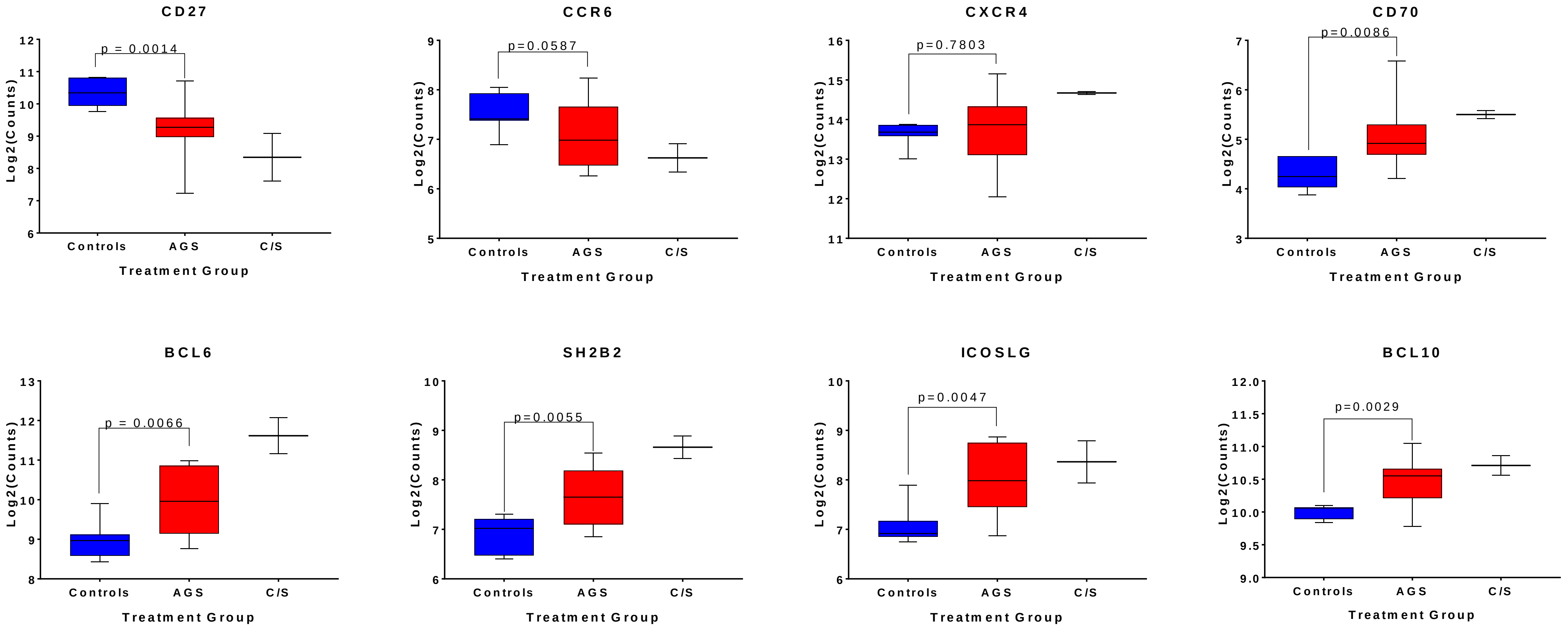
CD27
p = 0.0014
CCR6
p=0.0587
CXCR4
p=0.7803
CD70
p=0.0086
BCL6
p = 0.0066
SH2B2
p=0.0055
ICOSLG
p=0.0047
BCL10
p=0.0029
Log2(Counts)
Controls
AGS
C/S
Treatment Group

Figure 6.
